# Genetic disruption of serine biosynthesis is a key driver of macular telangiectasia type 2 aetiology and progression

**DOI:** 10.1186/s13073-021-00848-4

**Published:** 2021-03-09

**Authors:** Roberto Bonelli, Brendan R. E. Ansell, Luca Lotta, Thomas Scerri, Traci E. Clemons, Irene Leung, The MacTel Consortium, Tunde Peto, Alan C. Bird, Ferenc B. Sallo, Claudia Langenberg, Melanie Bahlo

**Affiliations:** 1 Department of Medical Biology, The University of Melbourne, Parkville, Victoria 3052 Australia; 2 Population Health and Immunity Division, The Walter and Eliza Hall Institute of Medical Research, Parkville, Victoria 3052 Australia; 3 MRC Epidemiology Unit, University of Cambridge, Cambridge, CB2 0SL UK; 4 The EMMES Corporation, Rockville, MD 20850 USA; 5 Department of Research and Development, Moorfields Eye Hospital NHS Foundation Trust, London, EC1V 2PD UK; 6 Department of Ophthalmology, Queen’s University, Belfast, BT7 1NN UK; 7 Inherited Eye Disease, Moorfields Eye Hospital NHS Foundation Trust, London, EC1V 2PD UK; 8 Department of Ophthalmology, Hôpital Ophtalmique Jules-Gonin, Fondation Asile des Aveugles, University of Lausanne, Lausanne, Switzerland

**Keywords:** Retinal disease, Mendelian randomisation, Metabolomics, GWAS, Serine

## Abstract

**Background:**

Macular telangiectasia type 2 (MacTel) is a rare, heritable and largely untreatable retinal disorder, often comorbid with diabetes. Genetic risk loci subtend retinal vascular calibre and glycine/serine/threonine metabolism genes. Serine deficiency may contribute to MacTel via neurotoxic deoxysphingolipid production; however, an independent vascular contribution is also suspected. Here, we use statistical genetics to dissect the causal mechanisms underpinning this complex disease.

**Methods:**

We integrated genetic markers for MacTel, vascular and metabolic traits, and applied Mendelian randomisation and conditional and interaction genome-wide association analyses to discover the causal contributors to both disease and spatial retinal imaging sub-phenotypes.

**Results:**

Genetically induced serine deficiency is the primary causal metabolic driver of disease occurrence and progression, with a lesser, but significant, causal contribution of type 2 diabetes genetic risk. Conversely, glycine, threonine and retinal vascular traits are unlikely to be causal for MacTel. Conditional regression analysis identified three novel disease loci independent of endogenous serine biosynthetic capacity. By aggregating spatial retinal phenotypes into endophenotypes, we demonstrate that SNPs constituting independent risk loci act via related endophenotypes.

**Conclusions:**

Follow-up studies after GWAS integrating publicly available data with deep phenotyping are still rare. Here, we describe such analysis, where we integrated retinal imaging data with MacTel and other traits genomics data to identify biochemical mechanisms likely causing this disorder. Our findings will aid in early diagnosis and accurate prognosis of MacTel and improve prospects for effective therapeutic intervention. Our integrative genetics approach also serves as a useful template for post-GWAS analyses in other disorders.

**Supplementary Information:**

The online version contains supplementary material available at 10.1186/s13073-021-00848-4.

## Background

### MacTel disease and previous GWAS study

Macular telangiectasia type II (MacTel [1]) is a rare (0.045–0.1% prevalence) degenerative eye disease affecting the macula [2, 3]. MacTel is bilateral and progressively affects visual acuity [4, 5], reading ability [6] and vision-related quality of life [7, 8]. A successful phase II clinical trial has recently been reported for an intravitreal encapsulated cell therapy implant, showing efficacy in slowing the disease progression [9]. However, less invasive, non-surgical and more economical therapies are lacking. Given the rarity of MacTel and its subtle clinical signs, requiring several ophthalmological diagnostic methods, MacTel has been largely under/misdiagnosed [4]. Hence, further insight into the genetic basis of MacTel is key for accurate diagnosis, identifying future therapies, developing predictive models for the disease and increasing prognostic accuracy.

We previously published the first genome-wide association study on 476 MacTel patients and 1733 controls [10–12], identifying and replicating five loci. A single nucleotide polymorphism (SNP) at locus 5q14.3 (rs73171800) showed the strongest association with the disease and was previously identified to be associated with the quantitative traits of retinal venular and arterial calibre [13, 14]. The other four loci, 1p12 (rs477992), 2q34 (rs715), 7p11.2 (rs4948102) and 3q21.3 (rs9820286), were implicated in glycine and serine metabolism [15, 16]. Importantly, loci 3q21.3 and 7p11.2 did not reach genome-wide significance, and the SNPs at locus 3q21.3 were only in proximity, but not in linkage disequilibrium (LD), with those associated with MacTel. We additionally measured and found significant depletion of serum serine, glycine and threonine in MacTel patients compared to controls. These data provided the first insight into the genetic complexity underpinning MacTel and highlighted the potential involvement of metabolic and vascular trait disturbances in disease aetiology.

Glycine and serine are involved in many fundamental biochemical reactions. Pathogenic variants in genes involved in serine and glycine synthesis lead to severe congenital disorders such as phosphoglycerate dehydrogenase deficiency (*PHGDH*, 601815) and glycine encephalopathy (*GLDC*, 605899). Glycine and serine can be synthesised from one another, from other metabolic compounds or obtained through dietary intake.

A recent study reported the co-occurrence of MacTel in patients with a rare neuropathy, hereditary sensory and autonomic systemic neuropathy type I, or HSAN1 (162400) [17]. This neuropathy results from pathogenic loss of function variants in the enzyme serine palmityltransferase, leading to the accumulation of neurotoxic deoxysphingolipids. A similar accumulation of deoxysphingolipids was demonstrated in MacTel patients but, unlike HSAN1, was attributed to systemic serine deficiency.

MacTel is often comorbid with type 2 diabetes (T2D) [18]. It is unknown whether traits such as metabolite abundances, T2D or retinal vascular calibre, now known to be associated with MacTel, are causative or merely associated with the disease for other reasons. Causation hypotheses are now routinely investigated using Mendelian randomisation (MR). MR borrows from instrumental variable analysis and assumes that if a disease is caused by a particular intermediate phenotype, and the latter is caused by genetic variants, then the same genetic variants should be also associated with the disease [19–21]. Genetic variants involved in retinal vascular calibre traits and T2D have been previously identified [13, 14, 22] and the largest metabolomic GWAS meta-analysis to date recently identified almost 500 loci that are associated with differences in 142 serum metabolite levels in humans [23, 24]. The results from these studies can be used to investigate causal drivers of MacTel susceptibility via the MR approach.

Progress in MacTel diagnosis and treatment is complicated by the lack of clear indicators of early stages and by the substantial inter-patient and interocular heterogeneity of retinal phenotypes. Apportioning genetic variation to separate retinal malformations has the potential to shed light on the different biological mechanisms via which each locus contributes to the disease. Performing joint genetic and retinal phenotypic data thus investigates whether different retinal abnormalities are consequences of common or largely independent biological perturbations.

**Fig. 1:**
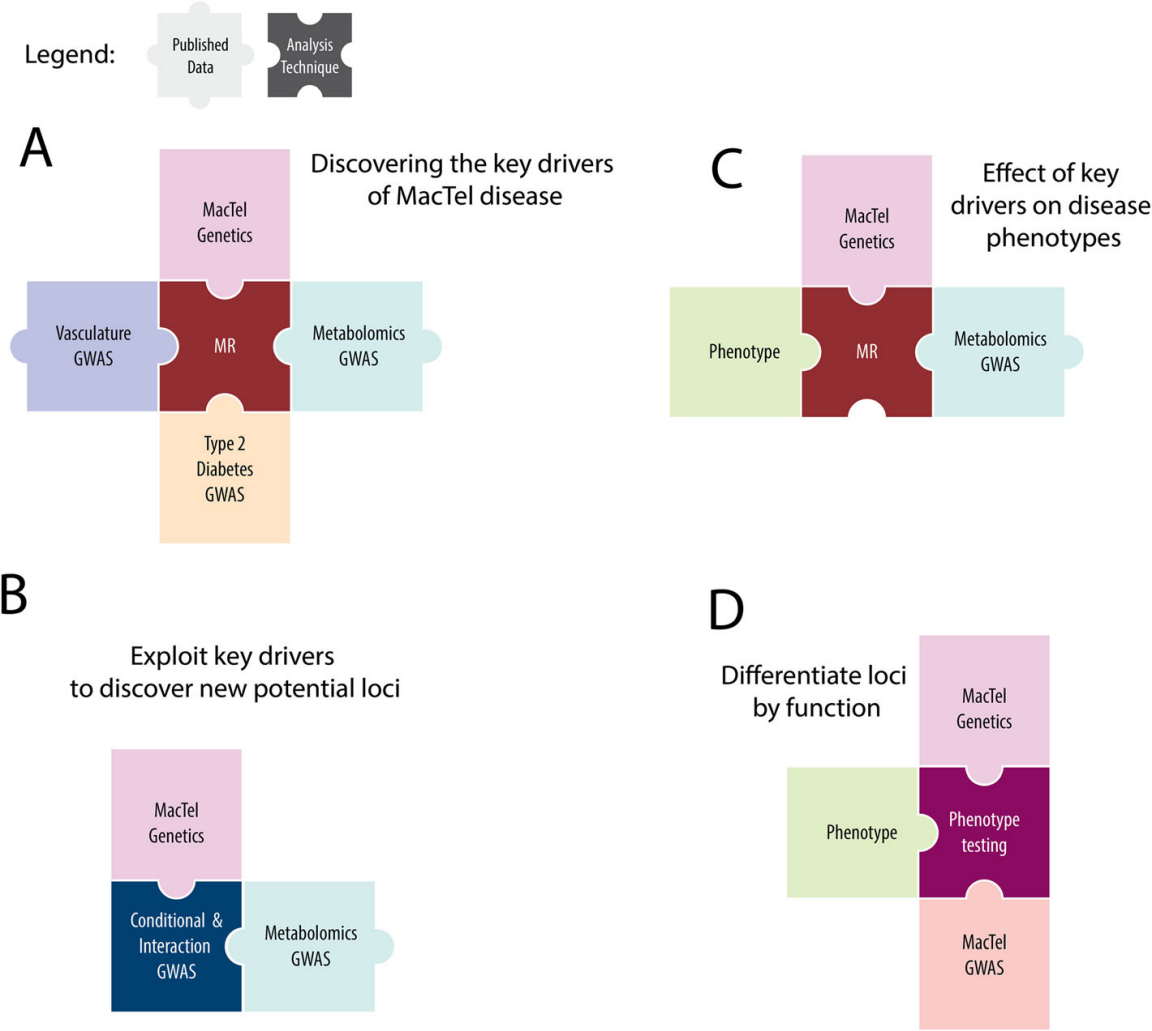
Study conceptual map. Each panel represents a study aim and depicts the data and the analysis technique used. Data is presented as pastel colour pieces with black typing while analysis techniques are presented as darker panels with white writing. MacTel genetics refers to individual-level SNP data available from our previous GWAS study. MacTel GWAS refers to the MacTel GWAS summary statistics. Vasculature GWAS refers to the summary statistics data available from two previous retinal vasculature calibre genetic studies [13, 14]. Metabolomics GWAS refers to summary statistics data available from a recently published metabolomics GWAS [23, 24]. T2D GWAS refers to summary statistics data available from GWAS study on T2D [22]

In this study, we test for the causality of several traits on MacTel disease (Fig. 1a). We exploit independent studies on MacTel-related traits to increase discovery power in our disease cohort, and both identify new disease loci while further resolving previously identified loci (Fig. 1b). We use extensive retinal phenotypic data from MacTel patients to identify key genetic drivers of specific subphenotypes (Fig. 1c). We collate genetic loci into functional groups by testing their combined effects on ocular spatial phenotypes (Fig. 1d). This study represents the first integrated analysis of MacTel genotypic data with MacTel-related traits as well as retinal imaging phenotypic data. Our study serves as a model for post-GWAS studies with phenotyping datasets.

## Methods

Unless otherwise stated, all statistical and computational analyses were performed using the R statistical software, version 3.5.1. False discovery rate (FDR)-corrected *p* values less than 0.05 were considered statistically significant. For genome-wide association analyses, uncorrected *p* values less than 5e−8 were considered genome-wide significant.

### Study participants

We recruited cases and controls at 23 participating clinical centres in seven countries (Australia, Germany, France, the UK, Switzerland, Israel and the USA). Informed written consent was obtained in accordance with the ethics protocols for human subjects approved by the appropriate governing body at each site in accordance with the Declaration of Helsinki as previously described [10]. Protocols and records of consent were centrally managed by the EMMES Corporation. The data was collected under IRB approved protocols, and all participants signed informed consents. A list of the ethics boards granting approval for human subject enrolment is available in Additional file 1: Supplementary Methods.

### DNA samples and SNP genotyping

Genotypic data was available for 476 MacTel patients and 1733 controls. SNPs were genotyped using the Illumina 5.0M chip as described previously [10]. Genetic predictors for metabolites and retinal vascular calibre were provided by the corresponding authors of each study [13, 14, 23, 24].

### Mendelian randomisation procedures

To perform Mendelian randomisation analysis on MacTel with metabolites, T2D and retinal vasculature, we used the allele score method for individual-level genetic data as described in section 3.1 by Burgess et al. [25]. This particular approach was selected because we have access to individual-level genetic data, our instruments were independent and instrument variable weights were externally and independently generated. For each metabolite and trait, only the top significant SNP at each genome-wide significant locus was used, with all SNPs independent of each other (no LD between SNPs within instruments). Using a *p* value threshold of 5e−8, we extracted SNPs significantly associated with serum metabolite concentrations or other traits of interest (retinal venular calibre, retinal arteriolar calibre and T2D). We estimated the metabolic polygenic risk scores (mPRS) and traits polygenic risk scores (tPRS) (Additional file 2: Table S1) for each subject by using the SNP magnitudes as weights (Additional file 1: Supplementary Methods). Using logistic regression models, we tested for the association between mPRS or tPRS and MacTel susceptibility. This analysis was corrected for genetically determined sex at birth and the first principal component to account for batch effects including population stratification, as in our previous publication [10]. We used Benjamini-Hochberg multiple testing correction to control the false discovery rate threshold of 5%. A conditional modelling approach was used to identify mPRSs independently associated with the disease. Specifically, the most significant mPRS was iteratively added as a covariate in the regression model until no mPRS was significant after FDR correction. The pleiotropic effect of the instrument variables used to create the mPRSs and tPRSs was assessed by performing the Egger test between the effect size of the instrument variables on both MacTel and the metabolites/trait risk [26]. Lastly, we generated visual representations of the correlation between the effect sizes for all significant traits to inspect the relationship for the robustness of the regression analysis, to ensure that there were no outliers driving the results.

### Conditional and interaction GWAS

Conditional GWAS were performed by including mPRSs or tPRSs in the logistic regression models as covariates. For the interaction GWAS, an interaction term was included for each SNP and the mPRS or tPRS combination of interest. Each model also included genetically determined sex at birth and the first principal component [10]. Analyses were performed using Plink v1.9 [27]. Results from the conditional and interaction GWAS were analysed using FUMA [28]. LocusZoom plots were produced using the LocusZoom software [29].

### Retinal phenotypes

We used longitudinal retinal phenotypic data on 1716 patients (3410 eyes) collected from Natural History Observation and Registry studies of Macular Telangiectasia [30]. The data consisted of 143 spatial measurements of retinal phenotypes (Additional file 3: Table S2). A detailed description of the corresponding methods can be found elsewhere [30]. Each phenotype was measured in 9 different sub-fields of the retina (defined by the ETDRS grid [31] Additional file 1: Figure S1). The phenotype data was cleaned by performing missing data imputation (Additional file 1: Supplementary Methods). The cleaned dataset contained 119 phenotypes that were collapsed into 30 biologically relevant endophenotypes using factor analysis (Additional file 1: Supplementary Methods).

### Investigating the relationship of retinal endophenotypes with genetic drivers

We tested for the association between retinal endophenotypes and significant disease-associated mPRSs, tPRSs and previously prioritised SNPs. The dataset containing retinal endophenotypes and genetic information included 3280 observations from 455 MacTel patients (907 eyes in total) with an average of 3.6 observations per eye over 10 years. Association testing was performed using a linear mixed model approach, assuming an additive effect of SNP alleles that increased MacTel risk. *p* values were corrected using an adaptive Benjamini-Hochberg procedure [32]. Further details are provided in Additional file 1**:** Supplementary Methods.

## Results

### Discovering key drivers of MacTel disease: metabolites

The MR procedure (Fig. 1a) applied to the metabolite panel highlighted 14 metabolic PRSs significantly associated with MacTel (Table 1, Additional file 4: Table S3). The two most significant metabolites were serine PRS and glycine PRS. In contrast to the metabolomics results from our previous metabolomics study with directly measured metabolites, threonine PRS was not significant. Other significant metabolites included arginine PRS, phenylalanine PRS and phosphatidylcholine species PRSs. Alanine PRS was borderline significant.

**Table 1:** Significant associations between mPRS and tPRS and MacTel. Regression coefficients are presented in the “Beta” column. Odds ratios (OR) are relative to a single standard deviation increase on the mPRS/tPRS scale. Odds ratios 95% confidence intervals are shown in the “95% C.I.” column. Benjamini-Hochberg-adjusted *p* values are presented under the “*p* value” column

Metabolite/Trait PRS	Beta	OR	95% C.I.	*p* value	Biochemical Family
Serine	−0.65	0.52	0.46–0.58	8.38E−28	Amino acids and biogenic amines
Glycine	−0.58	0.56	0.50–0.63	5.44E−19	Amino acids and biogenic amines
Lysophosphatidylcholine acyl C14:0	0.21	1.23	1.11–1.37	2.48E−03	Glycerophospholipids
Phosphatidylcholine diacyl C32:1	0.21	1.23	1.11–1.37	2.48E−03	Glycerophospholipids
Phosphatidylcholine diacyl C34:1	0.21	1.23	1.11–1.37	2.48E−03	Glycerophospholipids
Phosphatidylcholine with acyl-alkyl residue C38:1	0.20	1.22	1.10–1.36	2.97E−03	Glycerophospholipids
Arginine	0.17	1.18	1.07–1.31	2.83E−02	Amino acids and biogenic amines
Phenylalanine	−0.17	0.85	0.76–0.94	2.85E−02	Amino acids and biogenic amines
Arteriolar retinal calibre	0.32	1.38	1.25–1.52	2.22E−10	Vasculature trait
Venular retinal calibre	0.18	1.19	1.08–1.32	7.00E−04	Vasculature trait
T2D	0.17	1.19	1.07–1.33	7.72E−04	Comorbidity

The significant PRSs correlated with each other in our sample (Additional file 1: Figure S2), mirroring correlations observed with directly measured metabolite abundances. When all PRSs were re-tested in a model including serine PRS as a covariate, glycine PRS was the only associated metabolite (Additional file 4: Table S3). However, in this setting, glycine PRS significance decreased substantially (FDR = 0.022), while serine PRS remained strongly significant (FDR = 3.27e−11). No other metabolic PRS remained significant once serine PRS and glycine PRS had been included (Additional file 4: Table S3). Further, MR testing of a broader set of 248 metabolic PRSs abundances using results from Shin et al. [16, 33] (Additional file 2: Table S1) found a few additional significant univariate contribution from other metabolites (Additional file 1: Supplementary Materials and Additional file 5**:** Table S4). However, none remained significant after inclusion of serine PRS as a covariate.

### Discovering key drivers of MacTel disease: T2D and retinal vasculature

T2D PRS was significantly associated with MacTel, as were both arteriolar and venular calibre PRS when tested separately. However, the vasculature trait results were driven by a single SNPs: rs2194025 (for arteriolar calibre) and rs17421627 (for venular calibre), both in strong LD with SNP rs73171800 at 5q14.3 previously identified in our original GWAS [10]. When including both vascular traits in the same model, only arteriolar calibre remained significant, due to the reduced number of SNPs (*N* = 2) used to construct that PRS (Additional file 1: Figure S3). When PRSs for retinal arteriolar calibre and T2D were included in a model with glycine and serine PRSs, all remained significant.

**Fig. 2:**
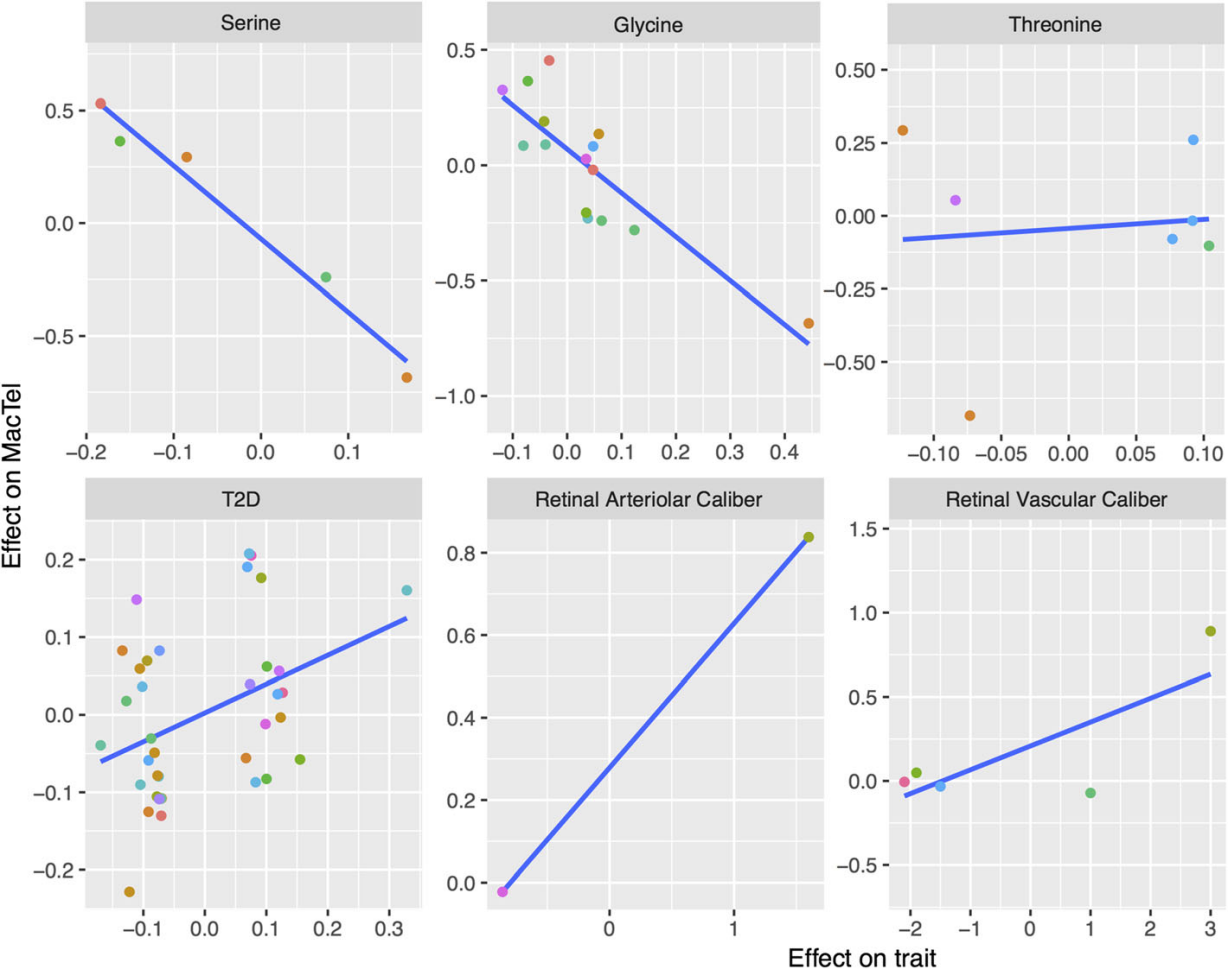
Effect size comparisons between MacTel and specific trait of SNPs used to define mPRS and tPRS. Each dot represents a SNP. Each panel represents a trait and contains the SNPs that were found to have a genome-wide significant effect on that trait. The *x*-axis captures the effect sizes of the SNPs on the trait while the *y*-axis captures the effect of the same SNPs on MacTel risk. The blue line represents a simple regression line between the two. Metabolites or traits causally affecting the disease are expected to have correlated effect sizes. Colour of the dots represents chromosomal positions where different chromosomes are represented by different colours

As a quality control analysis, we compared the effect sizes for each instrumental SNP on both MacTel and the metabolites or traits to inspect for potential SNP outlier effects of pleiotropy (Fig. 2). A negative, linear relationship was detected between the effect on the metabolic abundance and MacTel risk for the SNPs used to define glycine and serine. A positive relationship was observed for T2D and vasculature traits, while no relationship was detected for threonine which was used as a control trait, where no significant association was observed. We additionally observed that the regression line for all significant traits except the vasculature related traits, did not have intercept terms that were significantly different from 0 (b_0,serine_ = − 0.07, *p* = 0.2; b_0,glycine_ = 0.070, *p* = 0.131; b_0,T2D_ = 0.002, *p* = 0.88) suggesting a non-pleiotropic effect of the instruments used to test such traits [26] (Fig. 2). The same conclusion was drawn when glycine and T2D SNPs considered to have potential outlier effects were removed.

### Conditional and interaction GWAS

To uncover genetic correlates of MacTel independent of glycine and serine, we performed a GWAS conditioning on these mPRS (Fig. 1b). This analysis identified four genome-wide significant peaks (Additional file 1: Figure S4 a) and presented minimal inflation factor (1.029) (Additional file 1: Figure S4 e).

As expected, the original signal on locus 5q14.3 (rs73171800) related to vascular calibre remained significant, confirming the independence of this locus from genetic drivers of serine and glycine. A second genome-wide significant signal on locus 3p24.1 (rs35356316, *p* = 3.10e−08) was identified and was situated in a “gene desert” proximal to the genes *EOMES* and *SLC4A4*. Another single SNP in very close proximity to rs35356316 reached genome-wide significance as in the original MacTel GWAS study [10] but was believed to be a false positive, given the lack of LD with any other significant SNP (Additional file 1: Figure S4 b).

The remaining two conditionally significant SNPs tagged independent signals in locus 19p13.2 (Additional file 1: Figure S4 c-d). SNP rs36259 is an exonic non-synonymous SNP located in the *CERS4* gene which achieved close to genome-wide significance (*p* = 6.270e−08) and was nominally significant in our original study (*p* = 1.69e−7). In close proximity, we found an independent intergenic SNP rs4804075 (*p* = 3.72e−07) which lacked evidence of an eQTL effect on any neighbouring genes and which did not reach genome-wide significance. These results remain significant when conditioning on SNP rs73171800 (locus 5q14.3) and T2D PRS.

We performed additional GWAS analyses testing for interactions between all SNPs with serine, glycine, T2D PRSs and SNP rs73171800 (Fig. 1b). However, no further significant interacting loci were found (Additional file 1: Figure S5 a-d).

### Effect of key drivers on retinal endophenotypes

Given the small sample size of the endophenotypic data (*N* = 455), we only tested for the association between endophenotypes and glycine, serine and T2D PRSs, as these bore the clearest association with disease aetiology (Fig. 1c, Additional file 6: Table S5). No significant association between glycine or T2D PRSs and endophenotypes was found after correcting for serine PRS, and we, therefore, excluded them from additional testing. High serine PRS levels were found to be protective against loss of retinal transparency, leakage at the outer capillary network and the retinal pigment epithelium (RPE) in MacTel progression areas and leakage at the RPE in the MacTel area. We also found suggestive significance for protection from perivascular pigment clustering in the MacTel area and progression area. Higher serine PRS levels were associated with protection from macular thinning in the MacTel area and inferior inner area, and suggestive protection on nasal inner area and foveal area.

### Determining the effects of GWAS loci on endophenotypes

**Fig. 3:**
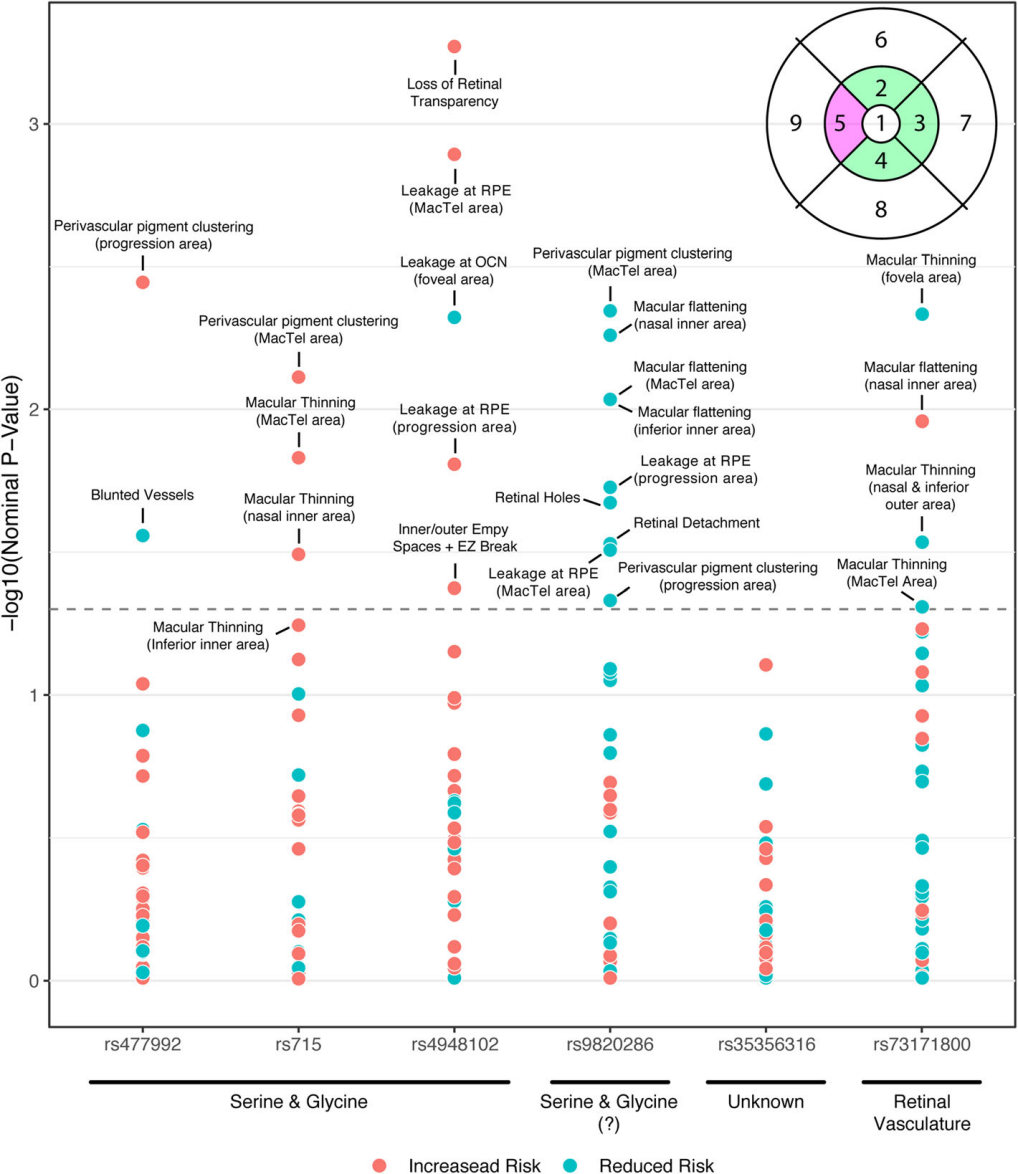
Association plot summarising all nominally significant association between MacTel SNPs and the endophenotypes (Additional File 6: Table S5). SNPs are divided into categories based on MR results. Each dot represents the nominal *p*-value for each association between all SNPs and endophenotypes. Red dots indicate that MacTel risk alleles for that SNP also increases the risk of the retinal phenotype, while blue dots indicate a reduced risk. The dashed line represents the nominal significance of *p* = 0.05. The top right corner displays the ETDRS grid for the right eye (OD) [31] used to divide the endophenotypes into 9 retinal areas: 1: foveal area, 2: superior inner area, 3: nasal inner area, 4: inferior inner area, 5: temporal inner area, 6: superior outer area, 7: nasal outer area, 8: inferior outer area and 9: temporal outer area. Although definitions vary across the literature, for simplicity, in this manuscript, we define the pink area as the “MacTel [onset] area” while the green areas are MacTel “progression areas”

To determine the likely functional impacts of the original GWAS loci, we tested for the associations with retinal endophenotypes (Fig. 1d, Fig. 3, Additional file 6: Table S5). No associations remained significant after correction for multiple testing due to the modest sample and effect sizes of individual SNPs on disease endophenotypes. Using a nominally (uncorrected) significant *p* value threshold denoted p* < 0.05 in an exploratory approach, we observed that locus 1p12 (rs477992) was associated with an increased risk of perivascular pigment clustering in the progression area, but protected against the risk of blunted vessels. Furthermore, locus 2q34 (rs715) increased the risk of macular thinning in the MacTel area and nasal inner area and the risk of perivascular pigment clustering in the MacTel area. Finally, the MacTel risk allele at locus 7p11.2 (rs4948102) increased the risk of retinal transparency, leakage at the RPE in both the MacTel area and progression area, as well as the presence of inner empty spaces and EZ break. This locus however protected against leakage in the outer layer of the foveal area.

These three loci, which converge on glycine/serine metabolism (1p12/rs477992/*PHGDH*, 2q34/rs715/*CPS1* and 7p11.2/rs4948102/*PSPH*), all demonstrated an overall susceptibility increment to RPE endophenotypes and other macular phenotype risks, lending further support for their role, despite not attaining significance after *p* value adjustment.

Interestingly, the MacTel risk allele at locus 3q21.3 (rs9820286) was only associated with protection from retinal disease endophenotypes. Specifically, locus 3q21.3 was associated with protection against retinal holes, retinal detachment, perivascular pigment clustering in the MacTel area and progression areas; leakage at the RPE level in the MacTel area and progression area; and foveal slope flattening in MacTel area, inferior inner area, and nasal inner area.

Locus 5q14.3 (rs73171800) corresponded to protection against macular thinning in the foveal area and nasal-inferior outer areas and increased risk of foveal flattening in the nasal inner area. Clustering the loci using hierarchical clustering on their regression coefficients (Figure S6) highlighted the unique endophenotype effect profiles of the 5q14.3 (rs73171800) and 3q21.3 (rs9820286) loci, and their distinctiveness in comparison with the other three loci, known to act on the serine/glycine pathway.

## Discussion

This study exploited well-powered publicly available GWAS data from traits of interest as well as deep retinal phenotyping data to further investigate the genetic aetiology of MacTel, a rare retinal disorder for which the first GWAS was only recently performed [10].

Mendelian randomisation and publicly available data were used to “genetically estimate” abundances of different metabolites. Although all PRSs in this study were generated using estimated SNP allele dosage effects from blood-based metabolomic studies rather than the retina, we believe that our results represent a useful approach for interrogating other retinal disorders.

We found serine depletion as the strongest causal driver of MacTel risk, with an association magnitude much greater than those observed when using single SNPs. The direction of the association between serine PRS and MacTel agrees with the from direct serum serine measurements reported previously [10], compelling additional evidence for a causal association between serine deficiency and MacTel. MacTel disease odds doubled for every standard unit decrease in serine PRS, and individuals in the lowest quintile of serine PRS presented an OR of 6.34 for MacTel compared to individuals in the top quintile. Our results also suggest a pronounced role of serine depletion in disease progression. As this analysis was applied only to MacTel patients and heterogeneity of retinal phenotypes may be under-represented, serine PRS was nevertheless able to partly discriminate between subjects with more advanced retinal abnormalities and those without. For example, lower serine PRS had an association with retinal greying in all retinal areas, a phenotype not observed in all MacTel patients [4]. Serine PRS also affected retinal thinning in the temporal parafoveal area, a marker of photoreceptor degeneration. Our results suggest a plausible biochemical explanation for differences in disease heterogeneity and progression. However, we acknowledge that our study used only aggregated retinal phenotypes and proxy measures of progression. Longitudinal data documenting progression is now required to confirm these findings. High concentrations of deoxy-sphingolipids, a byproduct of serine deficiency, have recently been shown to cause MacTel [17]. Our results provide evidence that genetically encoded serine depletion is causal for MacTel, which likely contributes to the disease by promoting deoxy-sphingolipid biosynthesis [17].

The metabolomics study performed by Lotta and colleagues [23, 24] has a sample size up to ~ 80 times greater than the MacTel discovery GWAS case sample size, although this varied by metabolite. With such a substantially greater sample size the metabolomics study was able to not only define the glycine and serine PRS but to also to compare their contribution to MacTel. Lotta and colleagues identified several pleiotropic SNPs that affect both glycine and serine abundance (4 out of 5 serine SNPs were also discovered to be genome-wide significant SNPs for glycine). This is likely due to the large correlation between these two metabolites. Previous metabolic studies have documented that glycine can be turned into serine and vice versa. When included along with serine PRS to predict MacTel, glycine PRS decreased substantially in significance. This suggests that the association between glycine PRS and MacTel may be in effect, due to the shared genetic signal between this metabolite and serine. We suspect that the glycine PRS remained significant in the conditional MR approach because it contained SNPs that were also associated with serine but were not discovered by Lotta and colleagues because of the smaller sample size available for this metabolite (2.5 times smaller). Lastly, the previously observed significant association of threonine to MacTel disease risk [10] through direct metabolite assessments in MacTel patient’s serum may be due to its metabolic dependence on glycine and serine, and thus a bystander effect.

SNP rs73171800 nominally affected macular thickness, specifically, a protective effect of the C allele against macular thinning, or an increase in macular thickness. Interestingly, a large GWAS study previously found that the G allele of SNP rs17421627—corresponding to the C allele of SNP rs73171800 (LD with rs73171800 *r*^2^ = 0.67)—was significantly associated with increased macular thickness [34]. A targeted study of SNP rs17421627 demonstrated enhancer activity which modifies the retinal vasculature [35]. Substituting the homologous zebrafish locus with a construct containing rs17421627 resulted in enhancer activity and changed expression of a proximal microRNA, mir-9-2 (homologous with human mir-9-5). Both mir-9-2 knock-down and endogenous enhancer knock-out animals showed dysmorphic retinal vasculature, indicating that rs17421627 may act on miRNA expression to modify the formation of the retinal vasculature in humans. Although we did not find a convincing causal relationship between retinal vascular calibre and MacTel, it may be that other features of the vasculature, for example, leakage, branching or integrity, are modified in rs17421627 carriers and account for the gross differences in macular thickness. Further, deep phenotyping of the retinal vasculature in these individuals may yield the physiological basis for this phenotype and its impact on MacTel.

We tried to increase discovery power compared to our previous work by conditioning on serine, glycine and T2D PRSs generated from much larger, independent studies. This exploratory analysis revealed two genetic contributors to MacTel which appear to be independent of the serine metabolic risk. These two loci were already found to be at least suggestively significant in our previous work, and as such, these loci should be considered as being further prioritised with this additional statistical evidence for the association. The locus 3p24.1, tagged by SNP rs35356316, lies between *EOMES* (604615) and *SLC4A4* genes (603345). Interestingly, *EOMES* encodes a transcriptional activator which is shown in mouse studies to interact with the retinal transcription factor *Pou4f2* [36]. The downstream gene *SLC4A4* encodes a sodium-coupled bicarbonate transporter which is expressed in Müller glia and the RPE, and functions to balance pH in the subretinal space [37]. Future GWAS with greater sample sizes should see both loci achieve genome-wide significance without requiring conditional analysis leveraging the PRS from other studies.

Conditional analysis revealed SNP rs36259 at locus 19p13.2 which tags an exonic, non-synonymous SNP located within the gene *CERS4* (615334), encoding a dihydroceramide synthase involved in sphingolipid biosynthesis [38]. Just as serine depletion is associated with defective sphingolipid synthesis, this locus may reduce sphingolipid production and thus contribute to MacTel.

Type 2 diabetics are overrepresented amongst MacTel patients [18]. We found a weak positive association between T2D PRS and MacTel risk. A possible explanation for this is that T2D involves major perturbations of patient metabolism. Specifically, a recent meta-analysis of metabolite abundances associated with pre-diabetes and/or T2D, found that glycine depletion tends to occur in this disease [39]. We show that glycine depletion is not likely to be causative for MacTel and is likely to be a consequence of metabolomic co-regularisation counterbalancing the genetically induced serine depletion. We speculate that diabetes may be a consequence rather than a cause of the metabolic phenotype underlying MacTel. Future MR studies may further dissect this interaction but will require a more highly powered MacTel GWAS. Indeed, T2D risk remained significant even after controlling for serine PRS and glycine PRS, which indicates the possibility of a separate mechanism unrelated to glycine/serine metabolism.

Our analysis revealed that locus 3q21.3, previously believed to act on the disease by modifying serum glycine and serine, is instead likely disconnected from these metabolites, as is the case for loci 3p24.1 and 5q14.3, implying that additional potential disease mechanisms exist.

## Conclusions

Our study demonstrates a likely causal contribution of serine abundance in both disease aetiology and progression. We additionally identified genetic contributors that are likely to act on disease development independently from serine availability. Future studies of disease progression and experimental validation of serine dependent and independent contributions will help to resolve the complex aetiology of MacTel and hence target treatments leading to increased efficacy.

## Supplementary Information

**Additional file 1:** **Figure S1.** Right (OD) and left (OS) eyes macula, were divided into 9 areas according to the ETDRS grid 1. 1: foveal area, 2: superior inner area (progression area), 3: nasal inner area (progression area), 4: inferior inner area (progression area), 5: temporal inner area (MacTel area), 6: superior outer area, 7: nasal outer area, 8: inferior outer area, and 9: temporal outer area. **Figure S2.** Correlation between mPRS that achieved multiple testing corrected significance (*p* < 0.05). Bigger dots and saturated colour indicate stronger correlations. Blue indicates a positive correlation while red indicates a negative one. **Figure S3.** Comparison between SNPs effect on vasculature traits and MacTel. Each dot is a SNP which was found to be significantly associated with a vasculature trait. The x-axis represents the effect that the SNP has on the particular trait while the y-axis displays the effect that the SNP has on MacTel. Most SNPs have no effect on MacTel. The only two SNPs that have an effect are the SNPs rs2194025 and rs17421627 which are very close to each other and in strong LD with SNP rs73171800 (r2=0.94 and r2=0.67). **Figure S4.** MacTel GWAS results conditioned by serine PRS and glycine PRS. A) Manhattan plot. B) Locus zoom plot of the significant signal on locus 3p24.1. C) Locus zoom plot of the significant signal by SNP rs36259 on locus 19p13.2. D) Locus zoom plot of the significant signal by SNP rs4804075 on locus 19p13.2. E) QQ-plot of association *p*-values. **Figure S5.** MacTel interaction GWAS results. A) Manhattan plot showing SNP interactions with serine PRS, B) Manhattan plot showing SNP interactions with glycine PRS, C) Manhattan plot showing SNPs interaction with SNP rs73171800 in locus 5q14.3, D) Manhattan plot showing SNP interactions with T2D PRS. **Figure S6.** Loci clustering heatmap. This clustering heatmap visualises the regression coefficients of each SNP on the endophenotypes. Rows and columns of the heatmaps were ordered according to the hierarchical clustering to give a visual representation of the similarities. SNP rs73171800 (5q14.3) and SNP rs9820286 (3q21.3) have very different effects from the other four SNPs which show a more consistent, shared pattern. **Table S7.** List of members and affiliations of the MacTel consortium researchers.

**Additional file 2:** **Table S1.** SNPs and weights used to create mPRSs, tPRSs, Shin et al mPRSs.

**Additional file 3:** **Table S2.** List of all phenotypes and measurement methods used to create the endophenotypes.

**Additional file 4:** **Table S3.** List of all mPRS associations and conditional mPRS association results.

**Additional file 5:** **Table S4.** List of all Shin et al mPRS associations results.

**Additional file 6:** **Table S5.** Endophenotypes analysis results.

**Additional file 7:** **Table S6.** Endophenotypes compositions and weights (available as .xlsx file). Phenotypes are presented in rows while Endophenotypes are presented as columns. The weight that each phenotype has on the endophenotype creation is presented in each cell value.

## Data Availability

The MacTel consortium genotypes for 678 samples (with consent) of the 704 samples genotyped as part of the discovery stage and that support the findings of this study have been deposited in the European Genome-phenome Archive (EGA), which is hosted by the European Bioinformatics Institute and the CRG, under accession EGAS00001002249 (https://www.ebi.ac.uk/ega/studies/EGAS00001002249) [11]. Controls genotype data from the project “NEI Age-Related Eye Disease Study (AREDS)–Genetic Variation in Refractive Error Substudy” is available on dbGap under accession phs000429, substudy of phs000001 (https://www.ncbi.nlm.nih.gov/projects/gap/cgi-bin/study.cgi?study_id=phs000429.v1.p1) [12].

## Abbreviations

MacTel: Macular Telangiectasia Type 2
GWAS: Genome-wide association study
SNP: Single nucleotide polymorphism
LD: Linkage disequilibrium
HSAN1: Hereditary sensory and autonomic systemic neuropathy type 1
T2D: Type 2 diabetes
MR: Mendelian randomisation
mPRS: Metabolic polygenic risk score
tPRS: Trait polygenic risk score
FDR: False discovery rate
Gly: Glycine
Ser: Serine
RPE: Retinal pigment epithelium
