# Supplementary material for: Genetic disruption of serine biosynthesis is a key driver of macular telangiectasia type 2 aetiology and progression
Source: Genome Med. 2021 Mar 9;13:39. doi: 10.1186/s13073-021-00848-4 (PMC7945323; doi:10.1186/s13073-021-00848-4)
Supplement: Supplementary file 1 — Additional file 1: Figure S1. Right (OD) and left (OS) eyes macula, were divided into 9 areas according to the ETDRS grid 1. 1: foveal area, 2: superior inner area (progression area), 3: nasal inner area (progression area), 4: inferior inner area (progression area), 5: temporal inner area (MacTel area), 6: superior outer area, 7: nasal outer area, 8: inferior outer area, and 9: temporal outer area. Figure S2. Correlation between mPRS that achieved multiple testing corrected significance (p < 0.05). Bigger dots and saturated colour indicate stronger correlations. Blue indicates a positive correlation while red indicates a negative one. Figure S3. Comparison between SNPs effect on vasculature traits and MacTel. Each dot is a SNP which was found to be significantly associated with a vasculature trait. The x-axis represents the effect that the SNP has on the particular trait while the y-axis displays the effect that the SNP has on MacTel. Most SNPs have no effect on MacTel. The only two SNPs that have an effect are the SNPs rs2194025 and rs17421627 which are very close to each other and in strong LD with SNP rs73171800 (r2=0.94 and r2=0.67). Figure S4. MacTel GWAS results conditioned by serine PRS and glycine PRS. A) Manhattan plot. B) Locus zoom plot of the significant signal on locus 3p24.1. C) Locus zoom plot of the significant signal by SNP rs36259 on locus 19p13.2. D) Locus zoom plot of the significant signal by SNP rs4804075 on locus 19p13.2. E) QQ-plot of association p-values. Figure S5. MacTel interaction GWAS results. A) Manhattan plot showing SNP interactions with serine PRS, B) Manhattan plot showing SNP interactions with glycine PRS, C) Manhattan plot showing SNPs interaction with SNP rs73171800 in locus 5q14.3, D) Manhattan plot showing SNP interactions with T2D PRS. Figure S6. Loci clustering heatmap. This clustering heatmap visualises the regression coefficients of each SNP on the endophenotypes. Rows and columns of the heatmaps were ordered according to [file 13073_2021_848_MOESM1_ESM.pdf]

Additional File1: Genetic Disruption of Serine Biosynthesis is a Key Driver of Macular Telangiectasia Type 2 Etiology and Progression

Additional Images:

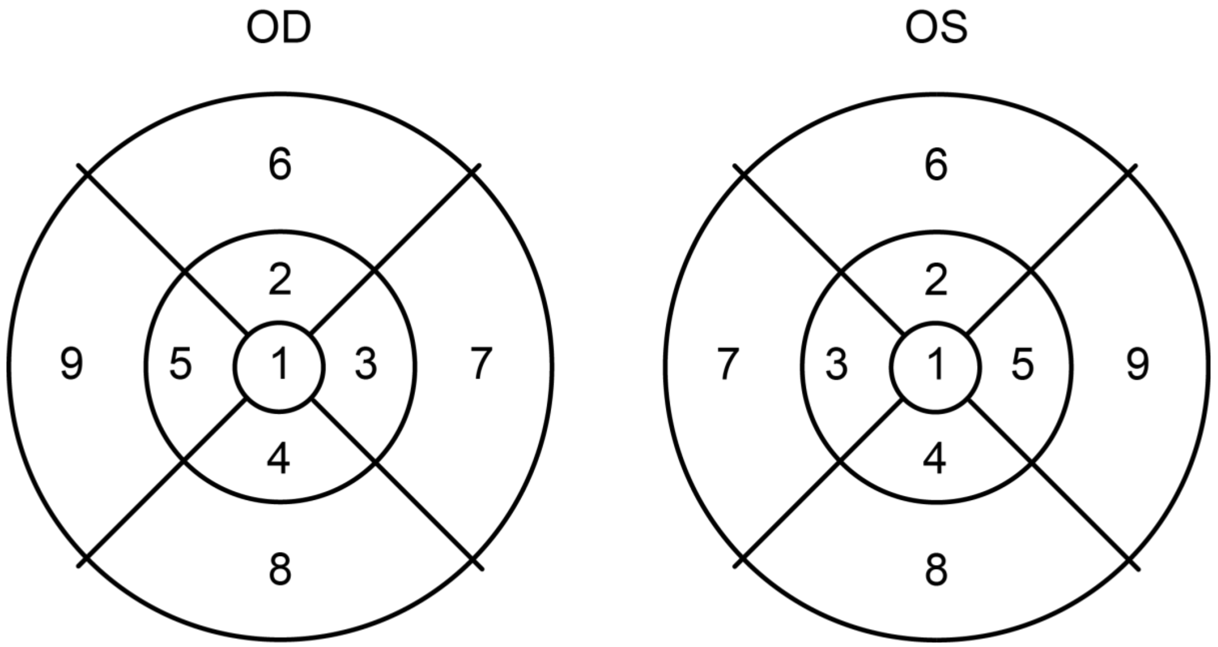

Fig S1: Right (OD) and left (OS) eyes macula, were divided into 9 areas according to the ETDRS grid [1]. 1: foveal area, 2: superior inner area (progression area), 3: nasal inner area (progression area), 4: inferior inner area (progression area), 5: temporal inner area (MacTel area), 6: superior outer area, 7: nasal outer area, 8: inferior outer area, and 9: temporal outer area.

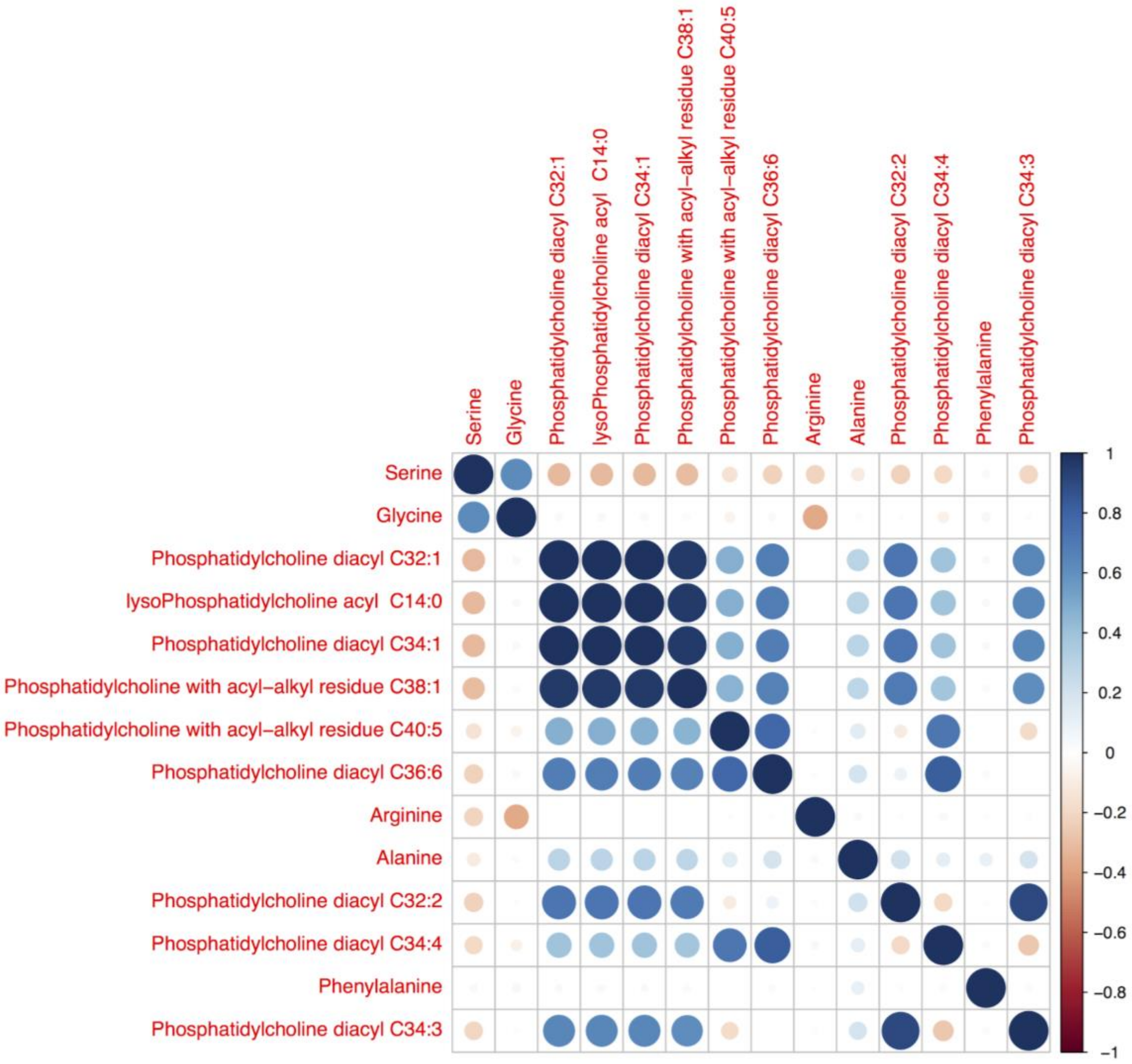

Fig S2: Correlation between mPRS that achieved multiple testing corrected significance ($p < 0.05$). Bigger dots and saturated colour indicate stronger correlations. Blue indicates a positive correlation while red indicates a negative one.

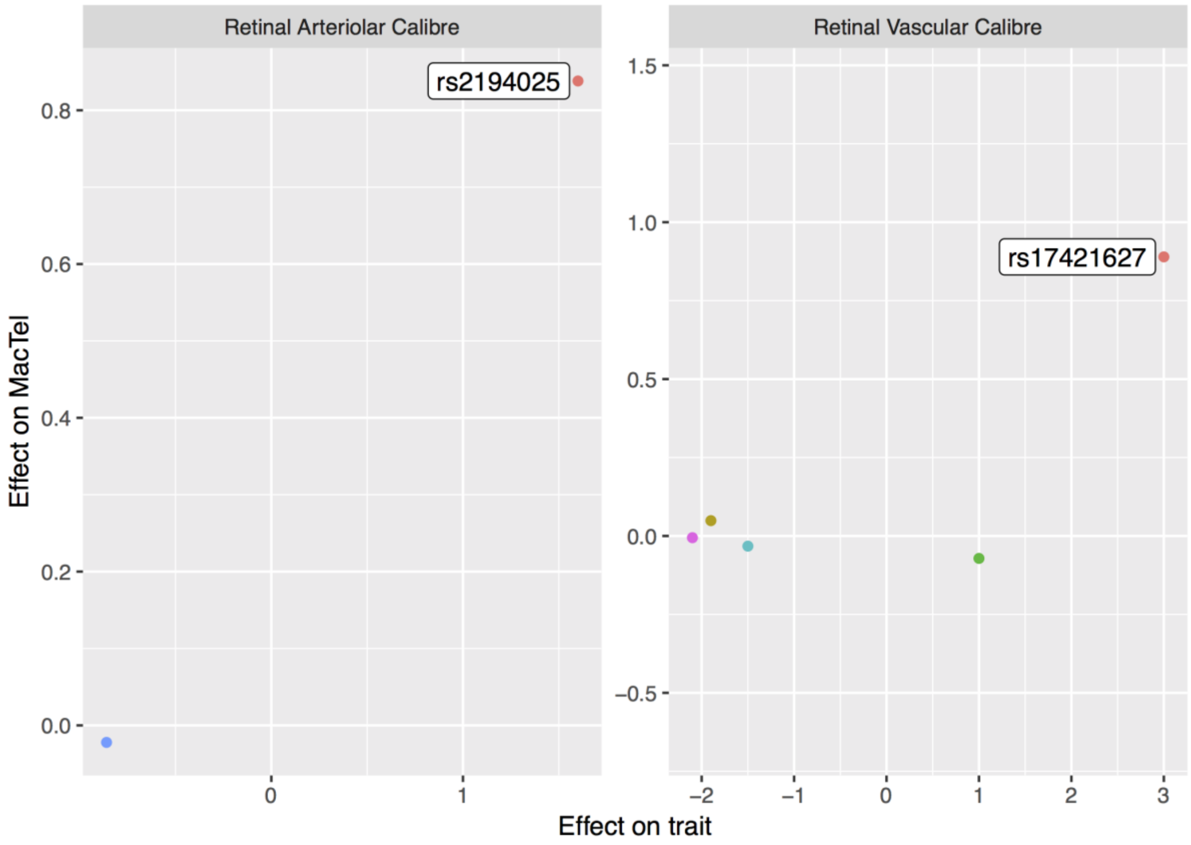

Fig S3: Comparison between SNPs effect on vasculature traits and MacTel. Each dot is a SNP which was found to be significantly associated with a vasculature trait. The x-axis represents the effect that the SNP has on the particular trait while the y-axis displays the effect that the SNP has on MacTel. Most SNPs have no effect on MacTel. The only two SNPs that have an effect are the SNPs rs2194025 and rs17421627 which are very close to each other and in strong LD with SNP rs73171800 (r^2=0.94 and r^2=0.67).

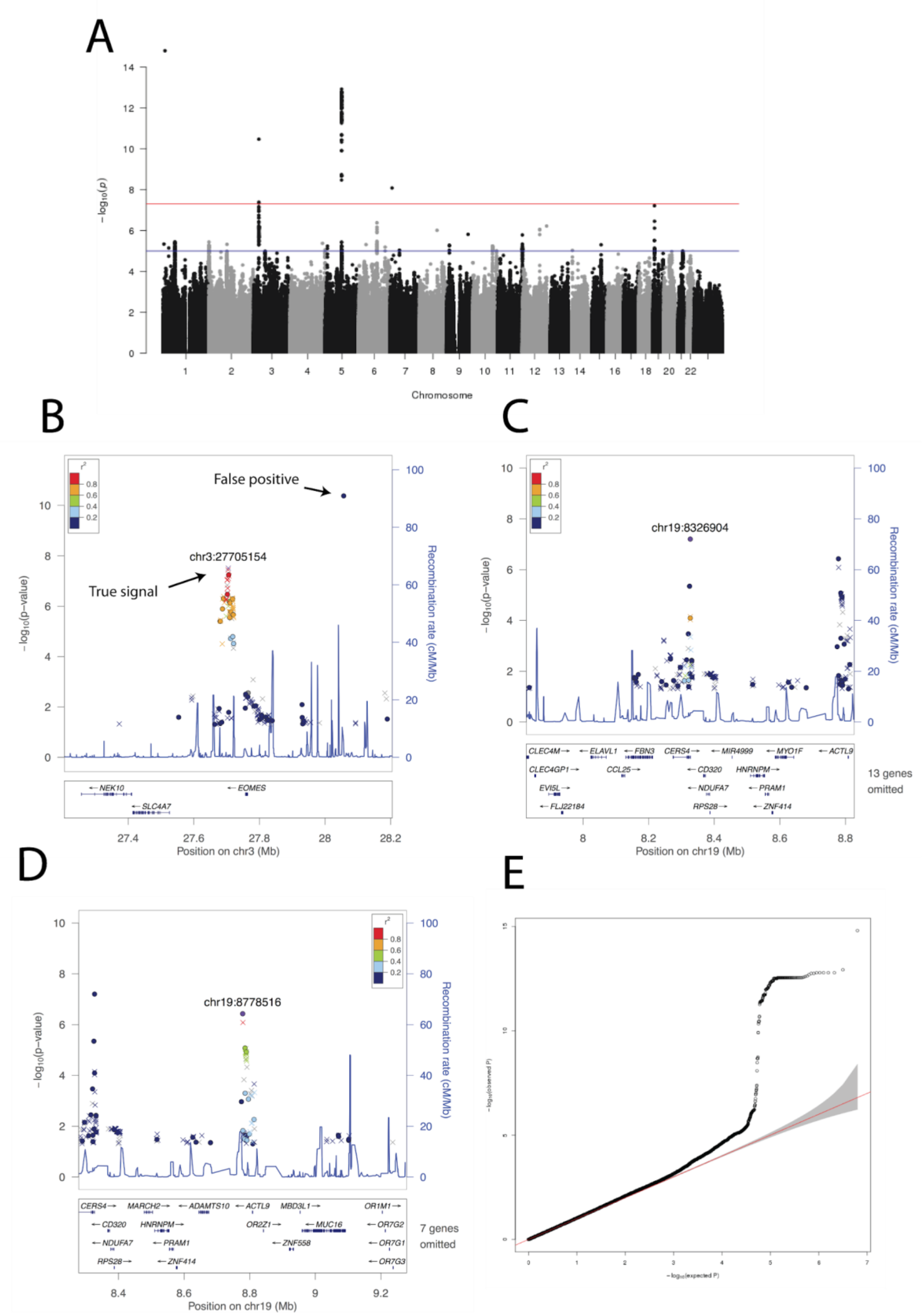

Fig S4: MacTel GWAS results conditioned by serine PRS and glycine PRS. A) Manhattan plot. B) Locus zoom plot of the significant signal on locus 3p24.1. C) Locus zoom plot of the

significant signal by SNP rs36259 on locus 19p13.2. D) Locus zoom plot of the significant signal by SNP rs4804075 on locus 19p13.2. E) QQ-plot of association p-values.

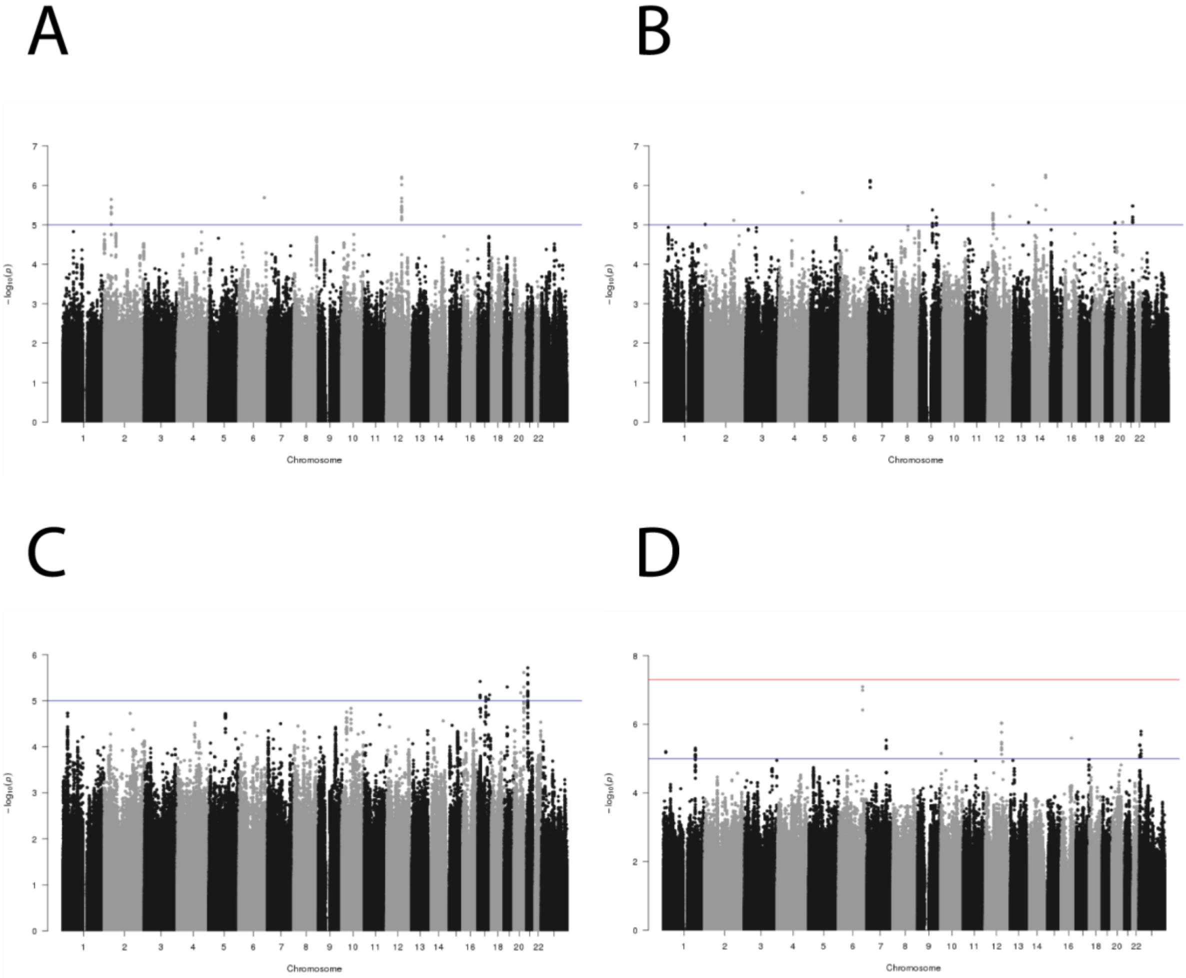

Fig S5: MacTel interaction GWAS results. A) Manhattan plot showing SNP interactions with serine PRS, B) Manhattan plot showing SNP interactions with glycine PRS, C) Manhattan plot showing SNPs interaction with SNP rs73171800 in locus 5q14.3, D) Manhattan plot showing SNP interactions with T2D PRS.

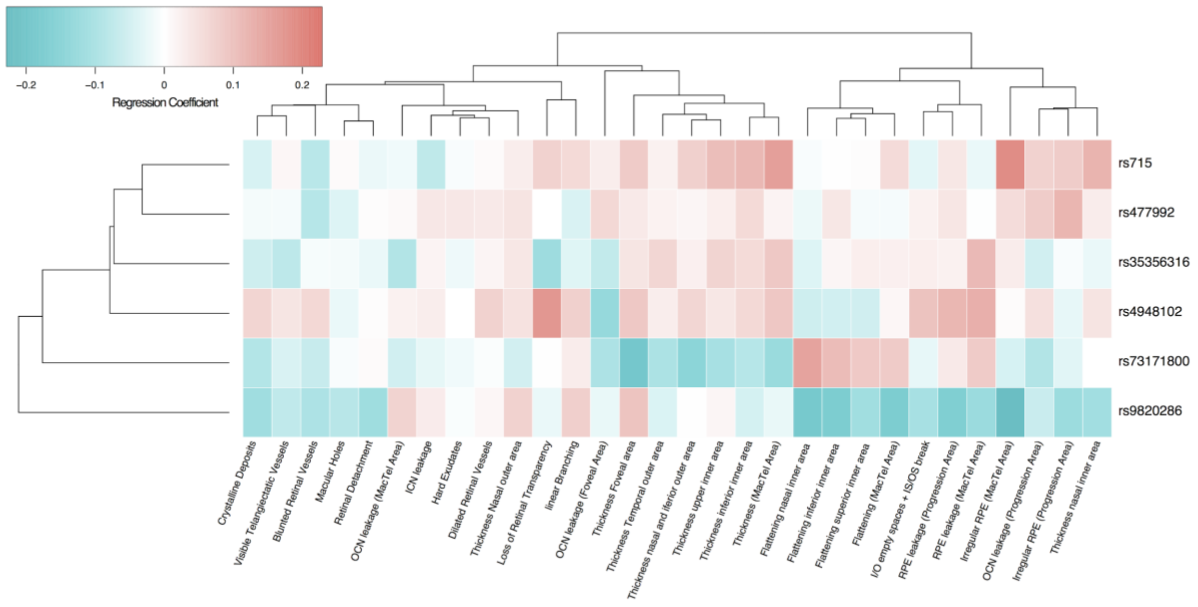

Fig S6: Loci clustering heatmap. This clustering heatmap visualises the regression coefficients of each SNP on the endophenotypes. Rows and columns of the heatmaps were ordered according to the hierarchical clustering to give a visual representation of the similarities. SNP rs73171800 (5q14.3) and SNP rs9820286 (3q21.3) have very different effects from the other four SNPs which show a more consistent, shared pattern.

Additional Methods:

Study Population

Our MacTel Project consortium (**Table S7**) recruited cases and controls at 23 participating clinical centers in seven countries (Australia, Germany, France, UK, Switzerland, Israel and United States). Informed written consent was obtained in accordance with ethics protocols for human subjects approved by the appropriate governing body at each site in accordance with the Declaration of Helsinki. Protocols and records of consent were centrally managed by the EMMES Corporation. The following ethics boards granted approval for human subject enrollment: Quinze-Vingts, Paris, France: Comité de Protection des Personnes Hôpital Saint-Antonie; Centre for Eye Research, Victoria, Australia: The Royal Victorian Eye and Ear Hospital; Clinique Ophtalmolgie de Creteil, Paris, France: Comité de Protection des Personnes Hôpital Saint-Antonie; Hospital Lariboisiere, Paris, France: Comité de Protection des Personnes Hôpital Saint-Antonie; Jules Stein Eye Institute, UCLA, California, USA: The UCLA Institutional Review Board; Lions Eye Institute, Nedlands, Australia: Sire Charles

Gairdner Group Human Research Ethics Committee; Manhattan Eye, Ear and Throat Hospital, New York, USA: Lenox Hill Hospital Institutional Review Board; Moorfields Eye Hospital, London, UK: National Research Ethics Service; Retina Associates of Cleveland, Inc., Cleveland, Ohio, USA: Sterling Institutional Review Board; Save Sight Institute, Sydney, Australia: South Eastern Sydney Illawarra Area Health Service Human Research Ethics Committee–Northern Hospital Network; Scripps Research Institute, La Jolla, California, USA: Scripps Institutional Review Board; St. Franziskus Hospital, Munster, Germany: Ethik-Kommission der Arztekammer Westfalen-Lippe und der Medizinishchen Fakultat der Westfallschen Wilhelms-Universitat; The Goldschleger Eye Institute, Tel Hashomer, Israel: Ethics Committee The Chaim Sheba Medical Center; The New York Eye and Ear Infirmary, New York, USA: The Institutional Review Board of the New York Eye and Ear Infirmary; The Retina Group of Washington, Olympia, Washington, USA: Western Institutional Review Board; University of Bonn, Bonn, Germany: Rheinische Friedrich-Wilhelms-Universität Ethik-Kommission; University of Chicago, Chicago, Illinois, USA: The University of Chicago Division of Biological Sciences–The Pritzker School Institutional Review Board; University of Michigan, Ann Arbor, Michigan, USA: Medical School Institutional Review Board (IRBMED); University of Wisconsin, Madison, Wisconsin, USA: Office of Clinical Trials University of Wisconsin School of Medicine and Public Health; The Wilmer Eye Institute of Johns Hopkins University, Baltimore, Maryland, USA: Johns Hopkins School of Medicine Office of Human Subjects Research; Scheie Eye Institute University of Pennsylvania, Philadelphia, Pennsylvania, USA: University of Pennsylvania Office of Regulatory Affairs; University of Bern, Bern, Switzerland: Kantonale Ethikkommission Bern; John Moran Eye University of Utah, Salt Lake City, Utah, USA: The University of Utah Institutional Review Board; Bascom Palmer Eye Institute University of Miami, Miami, Florida, USA: The University of Miami Human Subjects Research Office; Columbia University, New York, New York, USA: Columbia University Medical Center Institutional Review Board Category 4 waiver for research involving specimens obtained from deidentified subjects. Participants were given a standardized ophthalmic examination, including best corrected visual acuity, fundus photography, fluorescein angiography, optical coherence tomography and blue light reflectance. Images were adjudicated at the Reading Center at Moorfields Eye Hospital, London.

mPRS & tPRS calculation

We used the formula $PRS(M/T)_i = \sum_{j \in i} \; m_j * SNP_j$, where $PRS(M/T)_i$ is the polygenic risk score of metabolite (M) or trait, Ti. SNP_j is the jth SNP that was significantly associated with the metabolite or trait i. m_j is the magnitude (weight) that the SNP_j has on the metabolite or trait. The weight m_j was obtained as $m_j = \beta_j$ where β_j is the linear coefficient of the regression of SNP_j on the metabolite or trait i. We used a total of 499 SNPs to predict the abundances of 142 metabolites in blood serum, 37 SNPs to construct a genetically predicted T2D risk, 5 for retinal venular calibre and 2 for the retinal arteriolar calibre. mPRSs and tPRSs were normalized to have a mean of zero and unit standard deviation.

Imputation of phenotypic data

The phenotyping dataset contained multiple missing data points. Most missingness was related to imperfections in the ophthalmological images. By assuming missingness at random we imputed the missing data using predictive mean matching through chained equations, available in the package MICE [2].

Generation of Endophenotypes with Factorial Analysis

The phenotypic data was summarised into endophenotypes. Endophenotypes have been defined as "measurable components unseen by the unaided eye along the pathway between disease and distal genotype"[3]. To identify and construct these underlying endophenotypes while ensuring that each only contained phenotypes strongly related to each other, we used an iterative factorial analysis algorithm. Given the very different nature of the phenotypes observed we performed two separate analyses for the creation of the endophenotypes one to capture macular thickness endophenotypes, and the other to capture macular abnormality phenotypes.

Starting with 43 phenotypic measurements for macular thickness and 76 for macular abnormality phenotypes we reduced these two data sets further, by only retaining correlated phenotypes within each dataset.
We discarded all those phenotypes/variables which:

- Had an absolute value of Spearman correlation with all other variables less than 0.3.
- Had an absolute value of Spearman correlation with the overall dataset score of less than 0.3.
- Would decrease the Cronbach's alpha of the dataset

This procedure retained 33 phenotypes for macular thickness and 52 phenotypes for macular abnormalities. Variables that were retained after this step underwent an iterative factorial analysis procedure:

1. Perform a *parallel analysis* on the dataset to discover the number of factors F needed to summarise the phenotypic data
2. Run a *factorial analysis* with F number of factors
3. Rotate the factors using the *oblimin* rotation to allow for correlation between endophenotypes and obtain a loading value for each phenotype in each factor.
4. Create a sequence of loading thresholds T from 0.2 to 0.5 by 0.01
5. For each loading threshold $t \in T$ and for each factor $f \in F$, create a dataset that contains all the phenotype that have a loading greater than t. This results in F datasets.
6. Calculate the Cronbach's alpha in each dataset assessing the reliability of each factor.
7. Calculate the average of all factor's alphas
8. Select the loading threshold $t*$ that maximize the average alpha.

9. Eliminate from the dataset all the variables that do not have any loading greater than $t*$.
10. Eliminate all variables which create factors by themselves.
11. Repeat 1-10 the entire process until no variables are removed.

This procedure was followed by an additional step, where we took the phenotypes that were discarded from the previous step and re-introduced into the endophenotype creation process, by themselves. Those phenotypes that were discarded a second time, were manually collapsed into meaningful new endophenotypes from previous clinical knowledge. These endophenotypes were obtained by simply summing the phenotypes values together.

Each factor created by this process was considered an endophenotype in further analysis. All endophenotypes resulted in continuous variables for which higher values represented negative impacts on patient's eye health. Specifically, higher values of macular thickness endophenotypes represented a risk of macular thinning, higher values for foveal slopes endophenotypes represented increase risk of foveal flattening, and higher values for macular abnormalities endophenotypes represented an increased risk of observing that particular endophenotype. Most of the analyses to obtain the endophenotypes were performed using the R package Psych[4].

Following the method used by [5], we also created foveal slope measurements from each parafoveal area using the macular thickness endophenotypes. The formula used is was $Slope_j = tan^{-1}((Thick_j - Thick_1)/500)$. Where $Slope_j$ indicates the slope from the fovea to the parafoveal area $j$and$Thick_j$is the endophenotype of macular thickness in area j. Since the foveal slope is defined as the slope from the fovea to the parafoveal areas the only areas used were area 2, area 3, area 4, and area 5 of the ETDRS grid.

The factorial analysis results in 9 endophenotypes describing macular thickness and 19 describing macular abnormalities. We discarded one macular thickness endophenotype and one macular abnormalities endophenotype since they were believed to describe only a residual correlation between phenotypes and have no real clinical relevance. Additionally, we obtained 4 endophenotypes describing macular slope leading to a total of 30 endophenotypes to be used in the analysis.

Testing the identified disease key drivers and SNP loci against the endophenotypes

Endophenotypes were normalized to have zero average and unit standard deviation. Given the longitudinal nature of the data, we chose a linear mixed model approach to take into account the relationships within the subject and eye observation. Each model included all factors of interest as well as time defined as the number of days from the initial visit date, a quadratic function of visit year, gender, and age as covariates. Each model also contained a

random intercept and random time slope for each subject as well as a random intercept and random time slope for each eye within each subject. We included all these random effects since the log-likelihood of models that contained them and models which did not were significantly different (results not shown. Models that compared fixed effects were estimated using restricted maximum likelihood estimation. Given the large sample size, the p-values were obtained by assuming the asymptotic normality property of the t-values for each association. To take into account false discovery rate we used an adaptive Benjamini-Hochberg procedure [6]. We adopted this procedure rather than strict Bonferroni correction because of the very conservative nature of the linear mixed model and the small magnitude of SNP effect sizes.

Additional Results:

MR using Shin et al 2014 metabolomics study:

We also tried to perform MR testing on all metabolites available from one of the first studies on the genetic impact on metabolomics abundances by Shin et al 2014 [7,8] which contained many more metabolites than those initially available to us. Of the 248 mPRSs available we found few additional metabolites to be significant (Table S4), None of them was as strongly associated as glycine PRS and serine PRS and none retained significance after their inclusion as covariates.

Composition of the endophenotypes

A detailed list of phenotypes and respective loadings values used to estimate each of the endophenotypes is available in Supplementary Materials and **Table S6**. Interestingly, among the 30 retained endophenotypes, we noticed that some distinguished between abnormalities occurring in the initial MacTel region (area 5 in the ETDRS grid) and abnormalities expanding to the surrounding areas of the retina, which is usually denoted to indicate progression of the disease (areas 2, 3 and 4 on the ETDRS grid). For this reason, we defined these endophenotypes as "MacTel area" and "Progression" respectively.

Supplemental Table 7:

**The Emmes Corporation:**<br>***Coordinating Center***<br>401 N. Washington Street, Suite 700, Rockville, Maryland 20850<br>**Tel: 301.251.1161**<br><br>**MacTel@emmes.com**		
Sarah Duwel, RN MA CCRA<br>*Principal Investigator /*<br>*Project Director*<br>Tel: x10874	Traci Clemons, PhD<br>*Principal Investigator /*<br>*Statistician*<br>Tel: x10212	Clay Styer<br>*Data Manager*<br>*Advantage eClinical*<br>Tel: x 17345
Alexa Irazabal<br>*Administrative Coordinator –*<br>*Genetic Shipping Supplies*<br>Tel: x10860	Lena Bradley<br>*Project Manager – Lead CRA*<br>Tel: x10865	

**Lowy Medical Research Institute**<br>***Executive and Fiscal Sponsor***<br>L31, 85 Castlereagh Street Sydney, New South Wales, AU		
**Primary Contact:**<br>Jennifer Trombley RN MSN CCRC<br>*Director, Clinical Operations*<br>3366 N. Torrey Pines Ct., Suite 300<br>La Jolla, CA 92037<br>858.249.7109 office (US)<br>760.666.0452 mobile (US)<br>JTrombley@LMRI.net	Martin Friedlander MD PhD<br>*President – LMRI*<br>858.249.7100 office (US)<br>760.666.0452 mobile (US)<br>MFriedlander@LMRI.net	Anton Lever<br>*Group Manager, Finance*<br>AUS Tel: +612 9295 6015<br>AUS Mobile:<br>+614 0307 2143<br>alever@lfg.com.au

moorfields Reading Centre<br>*Moorfields Eye Hospital NHS*<br>*162 City Road*<br>*London EC1V 2PD*<br>MacTel@moorfields.nhs.uk	Columbia University, Genetics Lab<br>*Eye Institute Research Annex, Rm. 202*<br>*635 West 165th Street*<br>*New York, NY 10032*<br>*Tel: 212-305-8989*

Main contact:<br><br>**Konstantinos Balaskas**<br>**Consultant Ophthalmic Surgeon**<br>Konstantinos.Balaskas@moorfields.nhs.uk<br><br>**Administration:**<br><br>**Norma Jack**<br>**Tel: 020 7566 2815**<br>Norma.Jack@moorfields.nhs.uk	Main Contact:<br>**Carolyn Cai, Research Assistant**<br><br>Tel: 212-305-8501<br>Fax 212-305-7014<br>Cc3529@cumc.columbia.edu

Sites		
Site Name	Principal investigator	Coordinator
Centre for Eye Research (002)<br>Australia Macula Research Unit<br>Level 1, 32 Gisborne St.<br>East Melbourne VIC 3002	Robyn Guymer, PhD<br>Tel: 61 3 9929 8393<br>Fax: 61 3 9662 3859<br>Email:<br>rhg@unimelb.edu.au	Melinda Cain<br>Tel: 61 3 9929 8396<br>Email:<br>mcain@unimelb.edu.au<br><br>**EMILY CARUSO**<br>Tel: 61 3 9929 8113<br>Email:<br>emily.caruso@unimelb.edu.<br>au
Hôpital Lariboisière (004)<br>Service d'Ophtalmologie<br>2, rue<br>Ambroise<br>Paré,<br>Cedex 10<br>Paris<br>75475	Alain Gaudric, MD<br>Tel: 33 1 4995 6480<br>Fax: 33 1 4995 6484<br>Email: alain.gaudric@aphp.fr	Abir Zureik<br>Tel: 33 1 49 95<br>64 86<br>Fax: 33 1 49<br>95 64 83<br>Email: abir.zureik@aphp.fr

Jules Stein Eye Institute, UCLA (005)<br>100 Stein Plaza, Room 2-565<br>Los Angeles CA,<br>90095	Jean-Pierre Hubschman, MD<br>Tel: 310-206-5004<br>Fax: 310-825-3350<br>Email: hubschman@jsei.ucla.e	Rosaleen Ostrick<br>Tel: 310-794-5595<br>Fax: 310-794-5591<br>Email: ostrick@jsei.ucla.edu<br><br>Veronika Sadykov<br>Tel: 310-794-5596<br>Fax: 310-794-5591<br>Email:<br>Sadykov@jsei.ucla.edu<br>Mary Crowley<br>Tel: 310-267-5278<br>Email:<br>MECrowley@mednet.ucla.edu
Lions Eye Institute (006)<br>2 Verdun Street<br>Nedlands WA 6009	Ian Constable, MD<br>Tel: 61 8 9381 0882<br>Fax: 61 8 9381 0883<br>Email:<br>ian.constable@lei.org.au	Gareth Cooper<br>Email: GarethCooper@lei.org.au<br><br>Diana Bowman<br>Tel: +61 8 9381 0777<br>Fax: +61 8 9381 0700<br>Email: DianaBowman@lei.org.au
Vitreous, Retina, MACULA Consultants of New York (007)<br>460 Park Avenue<br>5th Floor<br>New York, NY 10022	Lawrence A. Yannuzzi, MD<br>Tel: 212-605-3777<br>Fax: 212-607-3795<br>Email:<br>layannuzzi@gmail.com	MAria Scolara<br>Email:<br>mscolaro@vrmny.com<br><br>Samira Shirwa<br>Tel: 212-452-6929<br>Email: sshirwa@vrmny.com<br>Sariah R. Persaud<br>Tel: 212-452-6929<br>Email:<br>sariahpersaud@vrmny.com

Moorfields Eye Hospital (008)<br>Clinical Trials Unit<br>162 City Road<br>London EC1V 2PD	Cathy Egan, MD<br>Tel: 44 20 7566 2262<br>Fax: 44 20 7608 6925<br>Email:<br>catherine.egan@moorfields.nhs.uk	Supeetha david<br>Tel: 0207 566 2835<br>Email:<br>Supeetha.David@moorfields.nhs.uk<br><br>Tjebo Heeren<br>Email:<br>Tjebo.Heeren@moorfields.nhs.uk
Retina Associates of Cleveland, Inc. (010)<br>3401 Enterprise Parkway, Suite 300<br>Beachwood, OH 44122	Lawrence Singerman, MD<br>Tel: 216-831-5700<br>Fax: 216-360-2196<br>Email: lsingerman@retina-assoc.com	Diane Weiss, RN<br>Tel: 216-831-5704 Ext. 244<br>Fax: 216-360-2196<br>Email: dew@retina-assoc.com
Save Sight Institute (011)	Mark Gillies, MD, PhD<br>GPO Box 4337 (use 2001 zip)<br>8 MacQuarie Street<br>Sydney NSW 2000<br>Tel: 61 4 1206 0313<br>Fax: 61 2 9382 7318<br>Email:<br>mark.gillies@sydney.edu.au	Stella Xu<br>Tel +61 2 9382 7309<br>Fax +61 2 93827278<br>Email:<br>stella.xu@sydney.edu.au<br>Sharon McKenzie<br>Tel +61 2 9382 7309<br>Fax +61 2 9382 7278<br>Email:<br>sharon.mckenzie@sydney.edu.au<br><br>Maria Williams<br>Tel +61 2 9382 7286<br>Fax +61 2 9382 7278<br>Email :<br>maria.williams@sydney.edu.au<br><br>Liza Shilpakar<br>Liza.shilpakar@sydney.edu.au

Scripps HEalth (012)<br>Scripps Clinical Research<br>10666 N. Torrey Pines Rd<br>La Jolla, CA 92037	Martin Friedlander, MD, PhD<br>Tel: 858-784-9138<br>Fax: 858-784-9135<br>Email: friedlan@scripps.edu	Jennifer Trombley, RN<br>Tel: 858-554-6678<br>Fax: 858-554-5016<br>Mobile: 760-492-6600<br>Email: Trombley.jennifer@scrippshealth.org
St. Franziskus Hospital (013)<br>St. Franziskus Hospital<br>Hohenzollernring 74<br>Muenster D-48145	Daniel Pauleikhoff, Prof. Dr<br>Tel: 49 251 933080<br>Email: dapauleikhoff@muenster.de	elke attenberger<br>Email: elke.attenberger@uveitis-zentrum.de<br>Tel: 0251-9352758<br>Martin Ziegler<br>Email: mpz@alex-ziegler.de
The Goldschleger Eye Institute (014)<br>The Chaim Sheba Medical Center<br>Tel Hashomer 52621	Joseph Moisseiev, MD<br>Tel: 972 3 534 3462<br>Email: Joseph.moisseiev@sheba.health.gov.il	Rinat Rashty<br>Tel: 972-3-530 2877<br>Email: Rinat.rashty@sheba.health.gov.il
The New York Eye and Ear Infirmary (015)<br>Department of Ophthalmology<br>310 East 14th Street<br>Suite 319 South Building<br>New York NY, 10003	Richard Rosen, MD<br>Tel: 212-979-4284<br>Fax: 212-979-4512<br>Email: rrosen@nyee.edu	**MELIZA UNSON**<br>Tel: 212-979-4672<br>Email: munson@nyee.edu
University of BONN (017)<br>Dept. of Ophthalmology<br>Ernst-Abbe-Str. 2<br>Bonn, 53127	Frank Holz, MD<br>Tel: 49 228 287 5647<br>Fax: 49 228 287 5603<br>Email: Frank.Holz@ukbonn.de	Simone Müller, Assistenzärztin<br>Email: Simone.Mueller@ukbonn.de<br><br>**Kerstin Bartsch**<br><br>Email: Kerstin.Bartsch@ukbonn.de

University of Michigan,<br>Kellogg Eye Center (019)<br>1000 Wall Street<br>Ann Arbor MI, 48105	Grant Comer, MD<br>Room 714<br>Tel: 734-763-5906<br>Fax: 734-936-2340<br>Email: gcomer@umich.edu	Pam Campbell, COT, CCRP<br>Room 1415<br>Tel: 734-936-0138<br>Email:<br>pamtitus@med.umich.edu
Scheie Eye Institute (022)<br>51 North 39th Street<br>Philadelphia PA, 19104	Alexander Brucker, MD<br>Tel: 215-662-8675<br>Fax: 215-662-8025<br>Email:<br>ajbrucke@mail.med.upenn.edu	Sheri Drossner<br>Tel: 215-662-8177<br>Fax: 215-662-8025<br>Email:<br>Sheri.Grand@uphs.upenn.edu
University of Bern (024)<br>Universitätsklinik für Augenheilkunde<br>Inselspital<br>Bern, CH<br>3010	Sebastian Wolf, MD, PhD<br>Tel: 41 (0)31 632 8503<br>Fax: 41 (0)31 632 4382<br>Email:<br>Sebastian.wolf@insel.ch	Corinne Stockli<br>Tel: 41 (0)31 632 1197<br>Fax: 41 (0)31 632 0114<br>Email:<br>Corinne.Stoeckli@insel.ch
University of Utah Health Care (025)<br>Moran Eye Center<br>University of Utah Health Care<br>65 Mario Capecchi Drive<br>Salt Lake City, UT 84132	Paul Bernstein, MD, PhD<br>Tel: 801-581-6078<br>Fax: 801-581-3357<br>Mobile: 801-560-3093<br>Email:<br>paul.bernstein@hsc.utah.edu	Barbara Hart<br>Email: Barbara.hart@hsc.utah.edu<br><br>Kara Halsey<br>Tel: 801-213-2034<br>Email: Kara.halsey@utah.edu<br><br>Kimberly Wegner<br>Tel: 801-584-6265<br>Email:<br>Kimberley.wegner@hsc.utah.edu<br><br>Sam Taylor<br>Email:<br>samclarktaylor@gmail.com<br><br>**ELIZABETH NUTTALL – Regulatory Documents**<br><br>Email:<br>Elizabeth.nuttall@hsc.utah.edu

Bascom Palmer (Miami) (026)	Philip Rosenfeld, MD, PhD<br>900 NW 17th Street<br>Miami FL, 33136<br>Tel: 305-326-6148<br>Fax: 305-326-6538<br>Email: prosenfeld@med.miami.edu	Cristina Lage-Rodriguez, MS, CCRC<br>1638 NW 10th Avenue, Suite 505B<br>Miami, FL 33136<br>Tel: 305-326-6117<br>Fax: 305-547-3662<br>Email: clage@med.miami.edu
Massachusetts Eye and Ear (027)<br>Retina Service<br>243 Charles Street<br>Boston, MA 02114	Joan Miller, MD<br>Tel: 617-573-3915<br>Fax: 617-573-3364<br>Email: joan_miller@meei.harvard.edu	Patricia Houlihan<br>Tel: 617-573-3646<br>Fax: 617-573-3514<br>Email: patricia_houlihan@meei.harvard.edu<br><br>Alex Leite<br>Email: Alex_Leite@meei.harvard.edu
Emory University (028)<br>Emory Eye Center<br>1365B Clifton RD NE<br>Atlanta, GA 30322	Jiong Yan, MD<br>Tel: 617-573-3915<br>Email: jyan2@emory.edu	Jayne Brown<br>Tel: 404-778-4430<br>Email: jmbrown@emory.edu
UNiversity of California – San francisco (029)<br>UCSF Department of Ophthalmology<br>10 Koret Way Box 0730, Room K209<br>San Francisco, CA 94143	JaCque Duncan, MD<br>Tel: 415-514-4241<br>Email: jacque.duncan@ucsf.edu	Arshia MiaN<br>Email: Arshia.Mian@ucsf.edu<br><br>MARY LEW<br>Email: Mary.Lew@ucsf.edu
University of Rochester (030)<br>Flaum Eye Institute<br>601 Elmwood Avenue Box 659<br>Rochester, NY 14642	Mina Chung, MD<br>Tel: 585-275-4141<br>Email: mina_chung@urmc.rochester.edu	Katherine Nedrow<br>Tel: 585-275-9485<br>Email:katherine_nedrow@urmc.rochester.edu

Medical College of Wisconsin (031)<br>The Eye Institute<br>925 N. 87th Street,<br>Room 807<br>Milwaukee, WI 53226	David Weinberg, MD<br>Tel: 414-955-2026<br>Email: dweinber@mcw.edu	Ellie Dorsey<br>Tel: 414-955-7854<br>Email: edorsey@mcw.edu<br><br>Shay Bourgeois<br>Tel: 414-955-2001<br>Email: sbourgeois@mcw.edu<br><br>B**rittany Rego**<br>Tel: 14-955-2031<br>Email: brego@mcw.edu
Radboud (033)<br>Radboud University Medical Center<br>Departments of Ophthalmology & Human Genetics<br>P.O. Box 9101, 6500 HB Nijmegen (409)	CB Hoyng, MD<br>Email: trialcentrum.ohk@radboudumc.nl<br>Tel: +31 (0)24 36 15255	Asha Kalisingh<br>Email: asha.kalisingh@radboudumc.nl<br>Tel: + 0243613212<br><br>chantal jumelet vanast<br>Email: Chantal.Jumelet-vanAst@radboudumc.nl<br><br>Clasien Oomen – regulatory<br>Email: clasien.oomenvanast@radboudumc.nl<br>Tel: + 31-24-36 15255
Oxford eye hospital (034)<br>John Radcliffe Hospital West Wing<br>Headley Way, Headington, Oxford, OX3 9DU	**Peter Charbel Issa MD, DPhil, FEBO**<br><br>T: 01865 227678<br>Email:<br>peter.charbelissa@ndcn.ox.ac.uk	Alexina Fantano<br>T: 01865 231053<br>F: 01865 231160<br>Email:<br>alexina.fantato@ouh.nhs.uk

Eye Center, Medical Center, Faculty of Medicine, University of Freiburg (035)<br>German: Klinik für Augenheilkunde, Universitätsklinikum Freiburg<br><br>UNIVERSITÄTSKLINIKUM FREIBURG<br>Klinik für Augenheilkunde<br>Studienteam der Sektion Retinologie<br>Dagmar Rommel<br>Studienassistentin / Studienkoordination<br>Killianstraße 5 · 79106 Freiburg /Germany<br>Telefon: 0761 270-40231<br>Telefax: 0761 270-86210	Felicitas Bucher, MD<br>Email: bucherf@scripps.edu	Dagmar Rommel<br>Email: dagmar.rommel@uniklinik-freiburg.de
Retina Research CEnter (036)<br>3705 Medical Parkway<br>Suite 420<br>Austin, Texas 78705	Chirag Jhaveri, Md<br>Email: cjhaveri@e-retina.net<br>Tel: 512-279-1251	ivana Gunderson<br>Email: igunderson@e-retina.net<br>Tel: 512-279-1251<br><br>Daniela Rodridguez<br>Email: drodriguez@e-retina.net
Retina Consultants of Southern Colorado (037)<br>2770 N. Union Blvd.<br>Suite 140<br>Colorado Springs, CO 80909	Ryan Rich, MD<br>Email: researchmdrich@coloradoretina.com<br>Tel: 719-473-9595	Jordan Townsend<br>719-473-9595 Ext. 123<br>Email: jtownsend@coloradoretina.com<br><br>Dragana Kuga<br>Email: dkuga@coloradoretina.com<br>Victoria Lee<br>Email vlee@coloradoretina.com<br><br>Kari Winger

		Email kwinger@coloradoretina.com
Cincinnati Eye Institute – CEI Physicians PSC, Inc (038)<br>1945 CEI Drive<br>Cincinnati, OH 45242	Daniel Miller, MD<br>Email: dmiller@cincinnatieye.com<br>Tel: 513-569-3669	Megan Kingdon, RN, BSM, CCRC<br>Email: mkingdon@cincinnatieye.com<br>Tel: 513-569-3669<br>Kelsi Dwyer<br>Email: kdwyer@cincinnatieye.com
University of Washington Eye Institute (039)<br>908 Jefferson St., 7th Floor, Seattle, WA 98104<br>Mailing Address: Box 359607, 325 Ninth Avenue, Seattle, WA 98104	Cecilia Lee, MD<br>Email: jung.cecilia@gmail.com	Ian Luttrell<br>Email: luttri@uw.edu<br>Francy Moses<br>Email: mosesf@uw.edumary
Byers Eye Institute - Stanford University (040)<br>2370 Watson Court<br>Palto Alto, California 94303	Diana Do, MD<br>Email: dianado@stanford.edu<br>Tel: 650-723-6995	Lisa Greer<br>Email: lgreer7@stanford.edu<br>Tel: 650-725-9184

MAyo Clinic (041)<br>200 First St SW<br>Rochester, MN 55905	Sophie Bakri, MD<br>Email:<br>bakri.sophie@mayo.edu	Heidi Rubin<br>Email:<br>Rubin.heidi@mayo.edu
Retina Center of New Jersey, LLC (042)<br>1255 Broad Street, Suite 104<br>Bloomfield, NJ 07003	Patrick Higgins, MD<br>Email:<br>Drhiggins@Retinacenternj.Com<br>Tel: 973-707-5632	Diane Deininger<br>Email:<br>ddeininger@retinacenternj.com<br>Tel: 973-707-5632<br>Fax :973-337-5865<br>Susan Anderson<br>Email:<br>sanderson@retinacenternj.com<br>Tel: 973-707-5632<br><br>Valerie Benavides<br>Email:<br>vbenavides@retinacenternj.com<br>Tel: 973-707-5632<br>KAthleen Rodriguez<br>Email:krodriguez@retinacenternj.com<br>Tel: 973-707-5632
Retina Associates New Orleans (043)<br>4315 Houma Boulevard Suite 201<br>Metairie, LA 70006	Stanislav A. Zhuk, MD, FACS<br>Email:stanislav.zhuk@retinaassociates.org<br>Tel: 504-456-9061<br>Fax: 504-895-8094	MElinda Mitchell<br>Email:<br>Melinda.mitchell@retinaassociates.org
Royal Oak (044)<br><br>Associated Retinal Consultants, P.C.<br>Neuroscience Center Building<br>3555 West Thirteen Mile Road Ste. LL-20<br>Royal Oak, MI 48073	Sandeep Randhawa	Kendra Mellert<br>E-mail: kmellert@arcpc.net<br>Tel: 248-288-9132 ext. 1313<br>Fax: 248-288-2265

Grand Rapids (144)<br><br>Associated Retinal Consultants, P.C.<br>1179 East Paris, S.E.; suite: 250<br>Grand Rapids, MI 49546	Paul V. Raphaelian, M.D.	Deb Markus<br>Email: dmarkus@arcpc.net
Traverse City (244)<br><br>Associated Retinal Consultants, P.C.<br>860 E. Front St.<br>Traverse City, Mi 49686	Scott Sneed	Serena Neal<br>Email: sneal@arcpc.net
Sierra Eye Associates (045)<br>950 Ryland Street, Reno, NV, 89502	Arshad Khanani, MD<br>Email: arshad.khanani@gmail.com<br>Tel: 775-329-0286	Deanna Dorman<br>Email:ddorman@sierraeyeassociates.com<br>Tel: 775-329-0286 x 141<br><br>**Heather Southwick**<br>Email: hpopovich@sierraeyeassociates.com
Retina Northwest – PORTLAND (046)<br>Portland<br>5440 SW Westgate Drive Suite 217<br>Portland, OR 97221	Michael Lee, MD<br>Email: MLee@retinanorthwest.com<br><br>Tel: 503-274-2121	Brian Puckett<br>Email: bpuckett@retinanorthwest.com<br>Tel: 503-274-2121<br>Fax: 503-229-8472
Eramus - Netherlands (047)<br>Department of Ophthalmology<br>Erasmus Medical Center Rotterdam<br>BA-130 postvak 'Trials'	J. Vingerling, MD<br>Email: j.vingerling@erasmusmc.nl	MARjo-Lan jansen<br>Email: m.jansen-vandrecht@erasmusmc.nl

3000 CA Rotterdam<br>The Netherlands		
New England Retina Consultants, PC (048)<br>3640 Main Street, Suite 201<br>Springfield, MA. 01107	David LaLLY, MD<br>Email: david.lally@gmail.com	Leah Persky<br>Email: persky.leah@gmail.com<br>Tel: 413-788-6044<br>Fax: 413-746-9715
CUMBERLAND VALLEY RETINA CONSULTANTS (049)<br>1150 Opal Court<br>Hagerstown, MD 21740	DAVID WARROW, MD<br>Email: Davidw@retinacare.net	Paula SMith<br>Email: paulas@retinacare.net<br>Tel: 301-665-1712<br>Fax: 301-655-1714<br><br>Katie Alvord<br>Email: katiea@retinacare.net<br>Tel: 301-665-1712 x 1024
Northwestern University (050)<br>259 E. Erie, Suite 15-20,<br>Chicago, IL 60611	Amani Fawzi, MD<br>Email: amani.fawzi@northwestern.edu	Regulatory:<br>Michell Kim<br>Email: michell.kim@northwestern.edu<br>Tel:312-503-2229<br><br>Nicole Seddon<br>Email: nreinhol@nm.org<br>Tel: 312.695-0252<br><br>Priya Thakkar<br>Email: Priya.Thakkar@nm.org<br>Tel: 312.695.2573

ZNA Middelheim Hospital (051)<br>Lindendreef 1<br>2020 Anterwerp, Belguim	Joke Ruys, MD<br>Email: joke.ruys@zna.be<br>Tel : +0032 3 280 3614	LiEsBeth VanPeene<br>Email: liesbeth.vanpeene@zna.be<br>Tel. +0032 3 280 3614
Bay Area Retina Associates (052)<br>122 La Casa Via Suite 223<br>Walnut Creek, CA 94598	Roger Goldberg, MD<br>Email:rgoldberg@bayareaetina.com	Farhan AhmeD<br>Email:fahmed@bayareaetina.com
Southeastern Retina Associates, P.C. (053)<br>1124 E. Weisgarber Rd. | Suite 207<br>Knoxville, TN 37909	Joseph M. Googe Jr, MD<br>Email: ctgooge@seretina.com<br>Tel: 865-588-0811	Kristina Oliver<br>Email: koliver@seretina.com
Mayo Clinic, Scottsdale Arizona (054)<br>13400 East Shea Boulevard<br>Scottsdale, AZ 85259	Odette Houghton, MD<br>Email: Houghton.Odette@mayo.edu	Michaele Menghini<br>Email: Menghini.Michaele@mayo.edu

Elman Retina Group, P.A. (055)<br>9114 Philadelphia Rd.<br>Suite 310<br>Baltimore, MD 21237	Michael J. Elman, M.D.<br>Email:<br>elman@elmanretina.com<br>Phone: 410- 686-3000<br>Fax: 410- 686-3690<br>Cell: 410-812-6440	JoAnn Starr<br>Email:<br>Starr@elmanretina.com<br>Phone: **410-686-3000**<br>FAX**: 410-686-3690**
Retina Consultants of Houston (056)<br>6560 Fannin Street<br>Suite 750<br>Houston, TX 77030	Charles Wykoff, MD, PhD<br>Email :<br>ccwmd@houstonretina.com<br>Phone: 713-524-3434	Kirsten Poldervart<br>Email:<br>kirsten.poldervart@houstonretina.com<br>Phone: 713-524-3434<br><br>Jose Munoz<br>Email:<br>jose.munoz@houstonretina.com<br>Phone: 713-524-3434<br>Cassandra ‘Cassie’ Cone<br>Email:<br>Cassandra.cone@houstonretina.com<br>Phone: 713-524-3434<br><br>MArie Chin (Regulatory)<br>Email:<br>marie.chin@houstonretina.com<br>Phone: 713-524-3434
Charles Retina (057)<br>1432 Kimbrough Road<br>Germantown, TN 38138	Steve Charles, MD<br>Email: scharles@att.net	Melanie Woods<br>Email:<br>mwoods@charlesretina.com
Tulsa Retina Consulants (059)<br>2424 E 21st Street,<br>Suite 200<br>Tulsa, OK 74114<br>918-794-6089	Lars Freisberg, MD<br>Email<br>Larsfreisberg@mac.com	Scott Hewitt<br>Email:<br>shewitt@retinatulsa.com<br>Phone: 918 -734-6344<br><br>Debbie Hardison<br>Email:<br>dhardison@retinatulsa.com

Palmetto Retina Center, LLC (061)<br>124 Sunset Court<br>West Columbia, SC 29169	JOHN A. Wells, III, MD<br>Email: jackwells@palmettoretina.com	Peggy Miller<br>Email: pmcdougal@palmettoretina.com<br>Phone: 803-404-5038<br>Fax: 803-744-0145<br><br>Mallie Taylor<br>Email: mtaylor@palmettoretina.com
Texas Retina Associates (062)<br>9600 N. Central Expressway, Suite 100<br>Dallas, TX 75231	Gary Fish, MD<br>Email: gefgef@ix.netcom.com	Karen Duignan<br>Email : kduignan@texasretina.com<br>Phone: 214-692-6885 x1<br>Fax: 214-265-0935<br><br>Tiffanie Keaton<br>Email: tkeaton@texasretina.com
Ophthalmic Consultants of Long Island (063)<br>360 Merrick Rd<br>3rd Floor<br>Lynbrook, NY 11563	Glenn Stoller, MD<br>Email : glstoller@gmail.com<br>Phone: 516-593-4026	Brennon Taylor<br>Email: btaylor@ocli.net<br>Phone: 516-593-4026<br><br>Susan Jones<br>Email: sjones@ocli.net<br>Phone: 516-593-4026<br><br>Carla Del Castillo<br>Email : cdelcastillo@ocli.net<br>Phone: 516-593-4026

Center for Retina and Macular Disease - Central Florida (065)<br>**250 Avenue K, SW**<br>**Suite 200**<br>**Winter Haven, FL 33880**	Richard Hamilton<br>Email: rich@crmd.net	Research Supervisor<br>Toni Collins<br>Email: tocol@crmd.net<br>Phone: 863-297-5400 Ext. 2046<br><br>Tammy<br>Email: tamc@crmd.net
**Retina-Vitreous Associates Medical Group (066)**<br>**9001 Wilshire Boulevard, Suite 301**<br>**Beverly Hills, CA 90211**	David Boyer M.D.<br>Email: vitdoc@aol.com	Janet Kurokouchi<br>Email: JKurokouchi@laretina.com<br>Phone: (310) 289-2478 x 3 (main study line)
**Global Research Foundation (067)**<br>**7447 N Figueroa Street**<br>**Los Angeles, CA 90041**	**Khaled Tawansy**<br>Email: ktawansy@globalresearchgroup.net	Research Supervisor<br>Monica Torres<br>Email: mtorres@globalresearchgroup.net<br>Phone: 323-257-3300 ext 120

References

1. Mathew R, Sivaprasad S, Florea D, et al. Agreement between time-domain and spectral-domain optical coherence tomography in the assessment of macular thickness in patients with idiopathic macular telangiectasia type 2. *Ophthalmologica*. 2013;230(3):144-150. doi:10.1159/000353455

2. Buuren S van, Groothuis-Oudshoorn K. mice: Multivariate Imputation by Chained Equations in R. 2011. http://doc.utwente.nl/78938/. Accessed March 10, 2017.

3. Gottesman II, Gould TD. The endophenotype concept in psychiatry: etymology and strategic intentions. *Am J Psychiatry*. 2003;160(4):636-645. doi:10.1176/appi.ajp.160.4.636

4. Revelle W. psych: Procedures for personality and psychological research. *Northwestern University, Evanston R package version*. 2014;1(1).

5. Jaisankar D, Raman R, Gondhale H, Chhablani J, Agarwal M. Visual function correlates of foveal slope changes on optical coherence tomography in macular telangiectasia type 2. *Retina*. December 2016. doi:10.1097/IAE.0000000000001416

6. Benjamini Y, Krieger AM, Yekutieli D. Adaptive linear step-up procedures that control the false discovery rate. *Biometrika*. 2006;93(3):491-507. doi:10.1093/biomet/93.3.491

7. metabolomics gwas server. http://metabolomics.helmholtz-muenchen.de/gwas/. Accessed December 4, 2018.

8. Shin S-Y, Fauman EB, Petersen A-K, et al. An atlas of genetic influences on human blood metabolites. *Nat Genet*. 2014;46(6):543-550. doi:10.1038/ng.2982
